# Therapeutic hypothermia achieves neuroprotection *via* a decrease in acetylcholine with a concurrent increase in carnitine in the neonatal hypoxia-ischemia

**DOI:** 10.1038/jcbfm.2014.253

**Published:** 2015-01-14

**Authors:** Toshiki Takenouchi, Yuki Sugiura, Takayuki Morikawa, Tsuyoshi Nakanishi, Yoshiko Nagahata, Tadao Sugioka, Kurara Honda, Akiko Kubo, Takako Hishiki, Tomomi Matsuura, Takao Hoshino, Takao Takahashi, Makoto Suematsu, Mayumi Kajimura

**Affiliations:** 1 Department of Biochemistry, Keio University School of Medicine, Tokyo, Japan; 2 Department of Pediatrics, Keio University School of Medicine, Tokyo, Japan; 3 JST Precursory Research for Embryonic Science and Technology (PRESTO) Project, Tokyo, Japan; 4 JST Exploratory Research for Advanced Technology (ERATO) Suematsu Gas Biology Project, Tokyo, Japan; 5 MS Business Unit, Shimadzu Corporation, Tokyo, Japan

**Keywords:** acetylcholine (ACh), carnitine, cerebral metabolism, hypoxia-ischemia (H-I), neonate, therapeutic hypothermia, quantitative imaging mass spectrometry (Q-IMS)

## Abstract

Although therapeutic hypothermia is known to improve neurologic outcomes after perinatal cerebral hypoxia-ischemia, etiology remains unknown. To decipher the mechanisms whereby hypothermia regulates metabolic dynamics in different brain regions, we used a two-step approach: a metabolomics to target metabolic pathways responding to cooling, and a quantitative imaging mass spectrometry to reveal spatial alterations in targeted metabolites in the brain. Seven-day postnatal rats underwent the permanent ligation of the left common carotid artery followed by exposure to 8% O_2_ for 2.5 hours. The pups were returned to normoxic conditions at either 38°C or 30°C for 3 hours. The brain metabolic states were rapidly fixed using *in situ* freezing. The profiling of 107 metabolites showed that hypothermia diminishes the carbon biomass related to acetyl moieties, such as pyruvate and acetyl-CoA; conversely, it increases deacetylated metabolites, such as carnitine and choline. Quantitative imaging mass spectrometry demarcated that hypothermia diminishes the acetylcholine contents specifically in hippocampus and amygdala. Such decreases were associated with an inverse increase in carnitine in the same anatomic regions. These findings imply that hypothermia achieves its neuroprotective effects by mediating the cellular acetylation status through a coordinated suppression of acetyl-CoA, which resides in metabolic junctions of glycolysis, amino-acid catabolism, and ketolysis.

## Introduction

Neonatal cerebral hypoxia-ischemia (H-I) evolving from acute perinatal events, such as placental abruption and severe fetal bradycardia, is a significant cause of neurologic disabilities and mortality in infants.^1^ A growing body of evidence indicates that the early initiation of mild hypothermia, *i.e.,* cooling the brain by as little as 3 °C to 6 °C, improves neurologic outcomes for full-term human infants after H-I.^2, 3, 4^ Rigorous investigation has been undertaken to identify control mechanisms.^5^ Nevertheless, the molecular mechanisms whereby cooling exerts its protective actions remain unclear. Thus, the goal of the present study is to define the biochemical mechanisms linking lowering temperature to the resulting neuroprotective effects.

Among several animal models, a model using 7-day postnatal rats developed by Vannucci's group^6^ has proven to produce an H-I brain injury consistently. The Vannucci model combines the unilateral ligation of a common carotid artery with a hypoxic insult. Although this unilateral-injury model has been extensively used in many laboratories, the biochemical alterations occurring in each hemisphere, which are fundamental to the evolution of neurologic derangements, have yet to be explicitly examined. Therefore, one of the aims of the current study was to characterize the dynamic changes in metabolic systems in ipsilateral (IL) and contralateral (CL) hemispheres for the first time.

Unlike hormonal signaling, where specific targets can be identified, cooling alters diverse functions of many macromolecules simultaneously, including enzyme activities and transport efficiency. This pleiotropic nature of the therapy makes it difficult to discern its precise actions. To characterize the alterations in metabolism induced by hypothermia systematically, recent investigations have used metabolic profiling of extracts from rat brain slices.^7^ However, such studies were unable to reveal spatial changes occurring in different regions of the brain, since the tissue homogenization process used to extract metabolites removes spatial information. To solve this problem, we used a series of advanced technologies that can offer ways to investigate cooling-responsive regulatory processes. First, we conducted a nontargeted metabolome analysis of brain tissues subjected to the presence or absence of hypothermia. Such profiling enables us to identify metabolic pathways responding to cooling treatment, thereby helping us to generate hypotheses.^8, 9^ Second, we conducted quantitative imaging mass spectrometry (Q-IMS) to examine not only the distribution patterns, but also the area-specific quantitative information for specific metabolites in the tissues.^10^ By doing so, we identified a common denominator in the effects of hypothermia: a reduction in acetyl-CoA content leading to a decrease in acetylcholine (ACh) and a concurrent increase in carnitine in specific regions of the brain, such as the hippocampus and the amygdala.

## Materials and methods

### Animal Model of Hypoxia-Ischemia

All animal procedures were conducted in accordance with the Animal Experimentation Guidelines of Keio University School of Medicine and were approved by the Laboratory Animal Care and Use Committee of Keio University (approval number: 10267). Male Sprague-Dawley rats (19 to 22 g, 7 days old, CLEA Japan, Tokyo, Japan) were used for the H-I model according to the method reported by Rice *et al.*^6^ Briefly, rats were anesthetized with isoflurane (5% for induction and 2% to 4% for maintenance) and a neck incision was made; the left common carotid artery was then ligated with 8-0 silk thread. After the incision was closed, and rats were allowed to fully recover from the anesthesia. Thereafter, the rats were returned to their dam for 2 to 4 hours to recuperate. The rats were then exposed to 8% O_2_ balanced with nitrogen (Nippon Megacare Corp., Tokyo, Japan) at 38°C for 2.5 hours (H-I group). Then, the animals were reoxygenated in room air for 3 hours while maintaining rectal temperatures either at 38 °C (normothermia group) or at 30°C (hypothermia group). Since these animals underwent a 5.5-hour nonfeeding period, a comparison was made against sham control animals that were not subjected to the H-I procedure but that were separated from their dams for the same duration at 38°C. Animals that were killed immediately after separation from their mother were designated as the normal group. During the H-I procedure, 11 out of 26 animals died (survival rate of 58%), but none died during the reoxygenation procedure. We analyzed all the animals that survived the procedure without exclusions for metabolome.

### Neurobehavioral Scores

**Figure 1:**
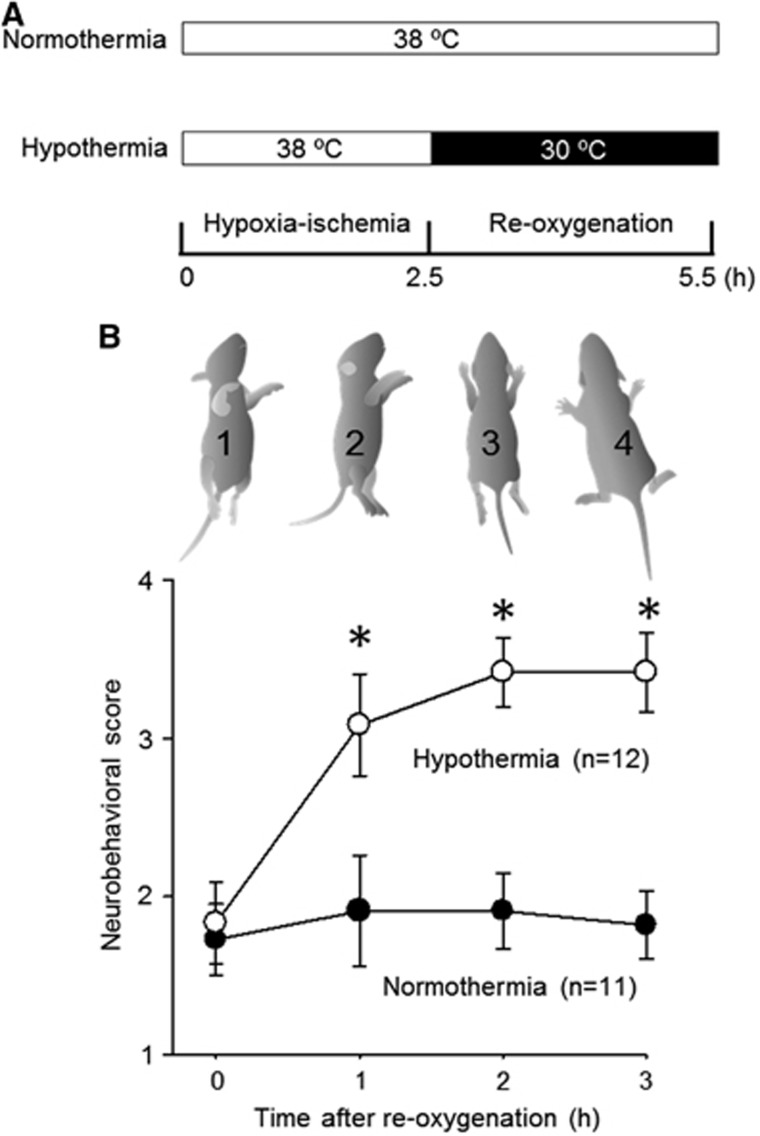
Hypothermia improves behavioral scoring. (**A**) Experimental protocol. Normothermia and hypothermia groups underwent the hypoxia-ischemia (H-I) (8% O_2_ with ligation of a left carotid artery) for 2.5 hours, followed by 3 hours of reoxygenation at 38°C and 30°C, respectively. The sham group remained in room air at 38°C for 5.5 hours. The pups were killed at the end of the reoxygenation period. (**B**) Behavioral scores were plotted against time after reoxygenation. The behavioral score (1 to 4) was assigned based on posturing, with 1 being the poorest and 4 being the best. Note that hypothermia improved the scores. *P<0.05, *versus* normothermia.

To confirm whether the hypothermic treatment was effective enough to achieve improved neurologic outcomes, as it does in clinical settings, we videotaped the animals during the reoxygenation phase and scored their neurobehavioral status at 0, 1, 2, and 3 hours after the start of reoxygenation. It should be noted that hypothermic procedure used in this study was found to accomplish prolonged improvements of behavioral scores even at 10 weeks after reoxygenation.^11, 12^ We used a 4-step neurologic scoring system to characterize the degree of injury severity based on the animals' posture, as shown in Figure 1: (1) a score of 1 was given for animals in a supine position (indicating the most severe degree of injury), (2) a score of 2 was given for those in a lateral decubitus position; (3) a score of 3 was given for those in a prone position with fully extended forearms; and (4) a score of 4 was given for those whose legs were in a prone position with flexed forepaws and legs (indicating the greatest degree of recovery). Scoring was performed in a blinded manner. Although our scoring system cannot detect the laterality; *i.e.*, more severe disabilities in right limbs resulted from the ligation of left carotid artery, it continuously gives us practical indices of physical strength without direct manipulation of the pups. This robust scoring showed an improvement after hypothermia treatment (Figure 1), confirming that our procedure was useful as a model for therapeutic hypothermia.

### Measurement of Brain Metabolites and Cluster Analysis

Capillary electrophoresis-electrospray ionization mass spectrometry (CE/ESI/MS) was conducted as previously described.^9, 10, 13, 14, 15^ Brain tissue is very prone to postmortem changes in labile metabolites. To minimize the autolytic alterations, the rats were submerged in liquid nitrogen immediately after cervical dislocation (<1 second) to lower the tissue temperature as rapidly as possible. Frozen brains were dissected from the skull with a surgical knife in the cryochamber (−30°C), halved and stored at −80°C until analyses. The frozen brains in vials were kept in the liquid nitrogen, taken out of vials, and quickly weighed with a microanalytical balance (<5 seconds) without thawing the tissue. They were then plunged into ice-cold methanol (500 *μ*L) containing internal standards (300 *μ*mol/L each of L-methionine sulfone for cations and MES for anions) and crushed using a tissue disruptor (Multi-Beads Shocker, Yasui Kikai, Osaka, Japan). Then, 250 *μ*L of ultrapure water (LC/MS grade; Wako, Osaka, Japan) was added, 750 *μ*L of the solution was transferred, and 500 *μ*L of chloroform was added, followed by thorough mixing. The suspension was centrifuged at 15,000 *g* for 15 minutes at 4°C. The upper aqueous layer was extracted again using chloroform and was centrifugally filtered through a Millipore 5-kDa cutoff filter (Millipore, Tokyo, Japan) to remove proteins. The filtrate was concentrated with a vacuum concentrator (SpeedVac; Thermo, Yokohama, Japan); this condensation process helps quantitate trace levels of metabolites. The concentrated filtrate was dissolved in 200 *μ*L of ultrapure water containing reference compounds (200 *μ*mol/L each of 3-aminopyrrolidine and trimesate) before the CE-MS analysis.

Quantification of the metabolites was performed using a capillary electrophoresis system (Agilent Technologies, Tokyo, Japan). It was equipped with an air pressure pump, a mass spectrometer (1200 series), an isocratic high performance liquid chromatography pump (G1603A CE/MS adapter kit), and a spray unit (G1607A). System control, data acquisition, and evaluation were conducted by commercial software for CE-MS (G2201AA Agilent ChemStation).

A hierarchical clustering algorithm included in Eisen's software^16^ was used to examine alterations in metabolite patterns among different groups. Comparative values (means±s.e.m.) for 107 metabolites' contents measured in five different groups are presented in Supplementary Table 1.

### Quantitative Imaging Mass Spectrometry

Quantitative imaging mass spectrometry was conducted according to a previously reported method,^10, 13, 15^ with some modifications. Coronal sections with a 10-*μ*m thickness were cut using a cryomicrotome (CM1900; Leica Microsystems, Tokyo, Japan) at a cryochamber temperature of −30°C. The sections were mounted on indium tin oxide-coated glass slide (either no. 578274, Sigma-Aldrich, St. Louis, MO, USA, or no. 237001, Bruker Daltonics, Billerica, MA, USA) kept at a temperature of −30°C. To aid the adherence of the sections, we briefly warmed the backside of indium tin oxide glass with the thumb. Adhered tissue sections were then freeze-dried in tubes containing silica-gel beads at −80°C.

Before matrix coating, the tissue slices were placed in desiccant for 10 minutes and allowed to equilibrate with room temperature. Freshly prepared 9-aminoacridine (25 mmol/L solution in 70% (v/v) ethanol) or 2,5-dihydroxybenzoic acid (50 mg/mL in 70% methanol and 0.1% trifluoroacetic acid), for the negative or positive ionization mode analyses, respectively, was sprayed over the samples using a Procon Boy FWA Platinum 0.2-mm caliber airbrush (Mr. Hobby, Tokyo, Japan). The samples were then subjected to laser scanning for MALDI-IMS or tandem MS (MS/MS) analysis.

Mass spectrometric imaging experiments were performed in the negative ionization mode for ATP, ADP, AMP, and glutamate and in the positive mode for carnitine using a MALDI mass spectrometer (AXIMA Performance, Shimadzu Corporation, Kyoto, Japan), and MS/MS analyses were performed with a prototype ‘Mass microscope' (Shimadzu Corporation).^17^ MALDI mass spectra were acquired using AXIMA Performance (Shimadzu Corporation) with 337-nm N_2_ laser, 10 Hz, operated in the reflectron mode. Unless otherwise noted, mass spectra were obtained with the following conditions: accelerating voltage of 20 kV, scanning mass range of 1 to 2,000 Da, and ‘Pulsed extraction mass' was set to 1,000 Da, respectively. The mass spectrometer was calibrated by an external calibration with spotted calibrants. In parallel, the laser power was optimized to obtain the high ratio of the standard to matrix molecule. The number of laser shots per spot was set to 100 to 200 with a roaming function that enables to obtain averaged data.

The MS/MS imaging for ACh was performed in the positive ionization mode using a MALDI-TOF/TOF MS (Autoflex III; Bruker Daltonics, Leipzih, Germany). The MS/MS imaging procedures were optimized according to a previously reported method,^18^ with some modifications. In the MS/MS imaging procedure, the data acquisition conditions, which included the laser power, collision energy, and number of laser irradiations, were optimized to increase the signal-to-noise (S/N) ratios of the target product ion for the fragment peaks. Relative-intensity maps were then constructed by flex imaging software 4.1 (Bruker Daltonics). The gray-scale images were generated by the digital imaging software (ImageJ, open source software, http://imagej.nih.gov/ij/), and were converted into numerical pixel-based data, allowing us to calculate apparent ACh contents ([ACh]_*app*_) as described below.

To construct apparent content maps for a specific metabolite, we modified a previously reported method.^10^ Since we could not harvest both a thick coronal section for the quantitative analysis and an adjacent section for the molecular ion-imaging study from the same animal, we used the mean values of metabolites measured in separate animals. This adaptation was unavoidable because the tissue sectioning of the neonatal brain was much more difficult than in adult animals as a result of the fragility of the neonatal frozen brain blocks arising from the high water content.^19^ This unique composition caused the brain tissues to break into pieces easily when we attempted to obtain 2-mm thick slices adjacent to the target position. Thus, the apparent content of a specific metabolite at the *i*th spot of tissue (*C*_*i*_) was estimated as follows:


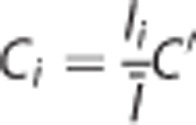


where *C*′ denotes the mean value of a metabolite content determined using CE/ESI/MS in CL hemispheres from several animals, *I*_*i*_ is the maximum intensity among the mass spectra within a specified range at the *i*th spot, and 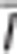 is the median of the maximum intensities of the metabolite from all the spots in the CL hemisphere.

### Statistics

All the values presented in this study were expressed as the mean±s.e.m. Differences between the means were evaluated for significance using an analysis of variance followed by the Fisher's exact test for multiple comparisons unless otherwise stated. Differences with *P*-values <0.05 were considered as statistically significant.

## Results

### Responses to Hypoxia-Ischemia on the Contralateral and Ipsilateral Hemispheres

**Figure 2:**
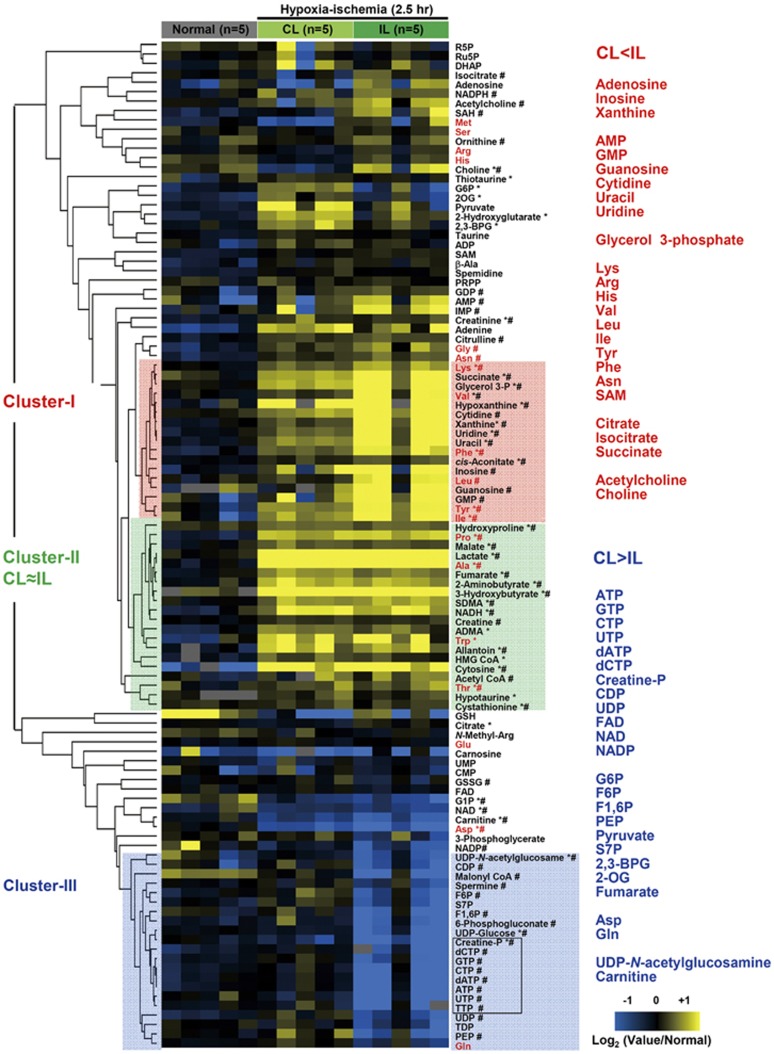
Responses to hypoxia-ischemia (H-I) in the contralateral (CL) and ipsilateral (IL) hemisphere. The hemispheric differences in the metabolic responses to H-I were categorized into three distinct clusters of metabolites: (i) enhanced in the IL (*red box*), (ii) unchanged between the CL and the IL (*green box*), and (iii) decreased in the IL (*blue box*). The fold changes were expressed as the log_2_ (H-I/normal) values. The metabolites next to the cluster tree in red represent standard amino acids. *P<0.05, H-I_*CL*_ *versus* normal. ^#^*P*<0.05, H-I_*IL*_ *versus* normal.

**Figure 3:**
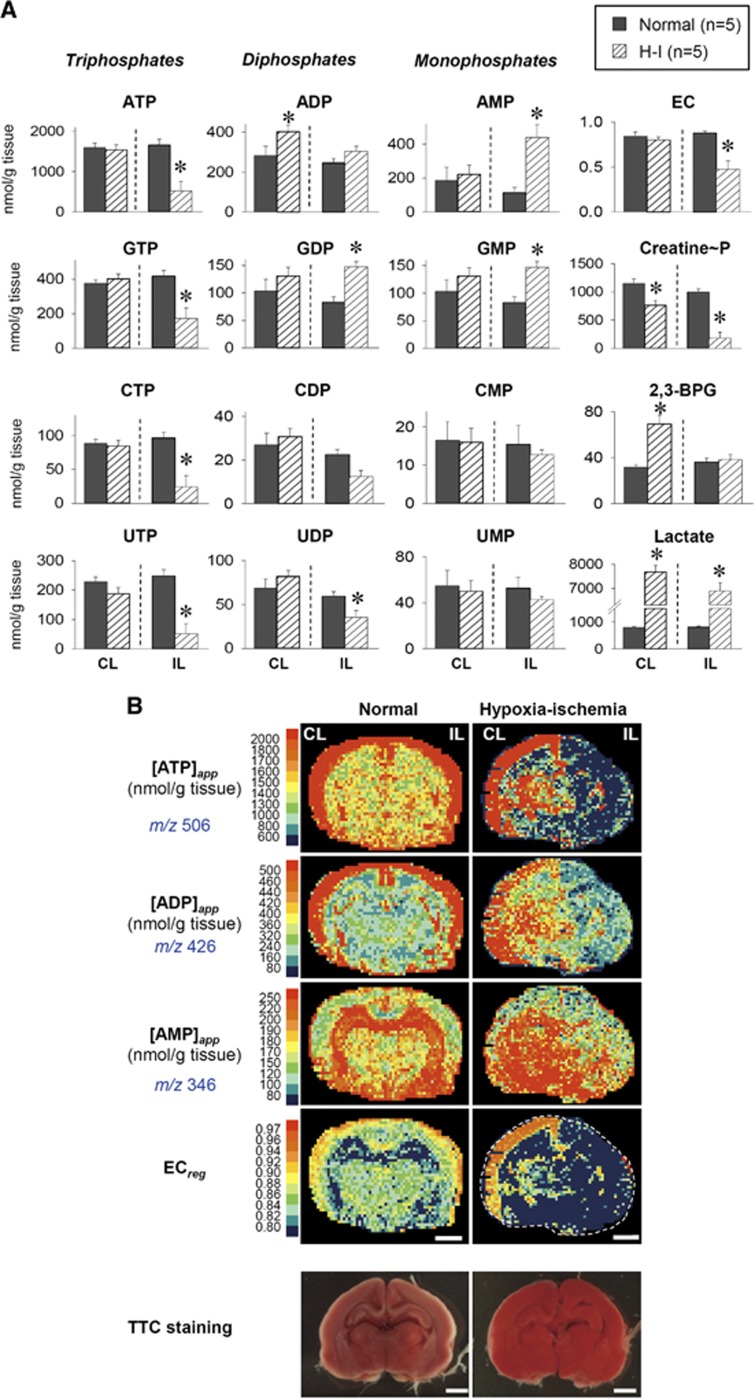
Changes in high-energy phosphates upon the hypoxia-ischemia (H-I) insult. (**A**) Quantification of high-energy phosphates and related metabolites by capillary electrophoresis-electrospray ionization mass spectrometry (CE/ESI/MS). Values are mean ±s.e.m. *P<0.05 compared with normal. CL, contralateral hemisphere; IL, ipsilateral hemisphere. (**B**) Quantitative imaging mass spectrometry (Q-IMS) showing spatial distributions of apparent contents of adenylates. Q-IMS analysis was conducted using a section with 10-*μ*m thickness. Content maps for ATP, ADP, and AMP were constructed on the same tissue. A map consists of ~40 × ~60 rectangles, each with an area of 0.2 × 0.2 mm^2^. Metabolic changes (*e.g.*, [ADP]_*app*_) were evident not only in the IL- but also in the CL hemispheres. Note the lack of an apparent infarction, as judged by the 2,3,5-triphenyltetrazolium chloride (TTC) staining conducted on separate animals (*bottom*). Scale bar=2 mm.

Since the Rice-Vannucci model of neonatal H-I involves the unilateral ligation of a common carotid artery,^6^ the severity of the insult is greater for the IL hemisphere than for the CL hemisphere, leading to different metabolic responses. Nevertheless, such hemispheric differences have not been systematically examined, except for high-energy phosphates and lactate.^20^ Therefore, we used a cluster analysis to compare and contrast the changes in 107 metabolites extracted from the two hemispheres at the end of a 2.5-hour H-I period. As seen in Figure 2, the analysis revealed three distinct clusters of metabolites: (1) Cluster I exhibited more pronounced increases in the IL than in the CL, (2) Cluster II exhibited no difference between the CL and the IL, and (3) Cluster III exhibited more pronounced decreases in the IL than in the CL. For nucleotide metabolism, increases in inosine, hypoxanthine, xanthine, and allantoin, the hallmarks of an ischemic response, were more pronounced in the IL than in the CL (Cluster I). High-energy phosphates in the IL decreased significantly, whereas those in the CL remained unchanged (Cluster III in Figures 2 and 3A). These results were consistent with those reported by Vannucci *et al.*^21^ The preservation of high-energy phosphates observed in the CL can be attributed to the increased consumption of glucose,^22, 23^ as evidenced, in part, by the surge in lactate which is the signature of enhanced glycolysis.

Hypoxia-ischemia caused increases in the majority of standard amino acids, with the exception of decreases in Asp and Glu. Of note, amino acids requiring molecular oxygen for their metabolism, namely, Tyr, Phe, Trp, and Pro, exhibited remarkable increases after oxygen deprivation. Contents of most amino acids in the IL were greater than those in the CL. In contrast, the IL contents of Glu, Gln, and Asp tended to be smaller than those in the CL (Figure 2, Supplementary Figure S1), suggesting that these glucose-derived amino acids are limited by the constrained blood flow in the IL.^22^ Furthermore, lipid-derived metabolites, including 3-hydroxybutyrate and glycerol 3-phosphate, were increased, suggesting that H-I induced a surge in lipolysis.

Energy consumption varies in different regions of the brain, with higher demand in the cerebral cortex compared with the hippocampus.^24^ We therefore examined spatial changes in adenylates in response to an H-I insult using Q-IMS (Figure 3B). In the normal brain, apparent contents of ATP and ADP ([ATP]_*app*_ and [ADP]_*app*_) were higher in the cortex than in other regions. However, those of AMP ([AMP]_*app*_) were higher in the hippocampus. A 2.5-hour H-I insult caused a mild reduction in values of subcortical [ATP]_*app*_ in the CL, while it substantially depleted [ATP]_*app*_ in the whole IL-hemisphere. It is worth noting that such region-specific biochemical alterations cannot be detected by the staining with 2,3,5-triphenylterazolium chloride, a conventional method to delineate the infarction at this acute phase of ischemia (<3 hours).

### Differential Responses of Energy and Amino-Acid Metabolisms to Hypothermia on the Contralateral and Ipsilateral Hemispheres

**Figure 4:**
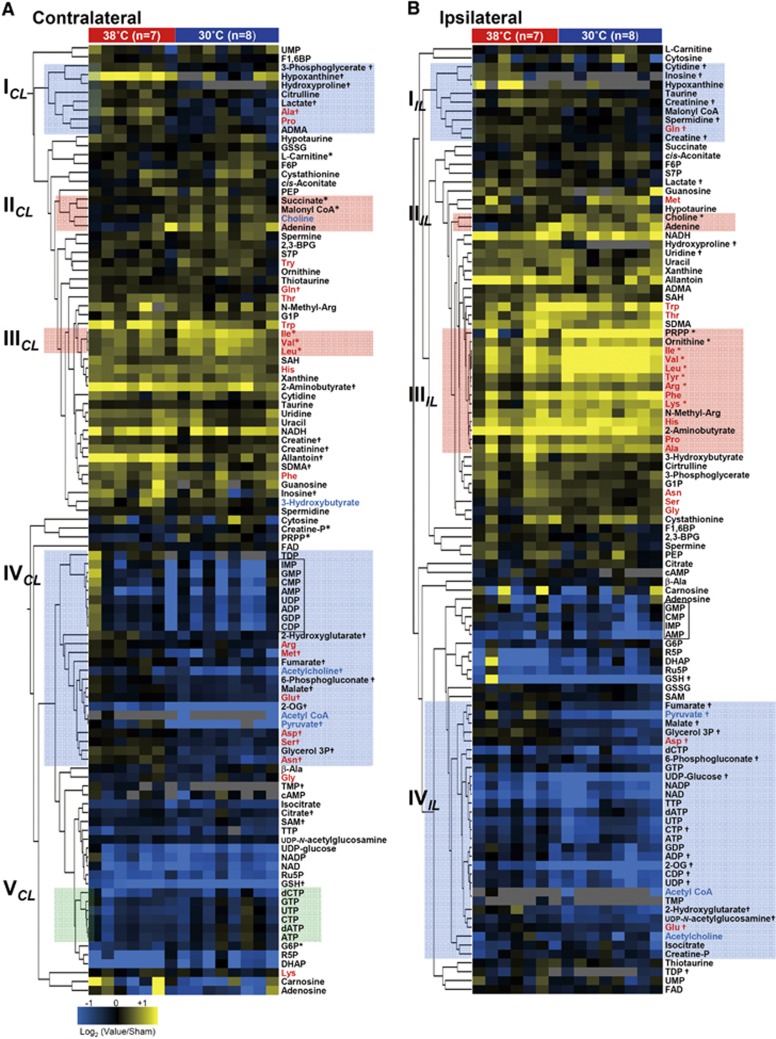
Identification of biochemical pathways responding to therapeutic hypothermia based on metabolic profiling. Metabolic changes induced by hypothermia in the contralateral (**A**) and ipsilateral (**B**) hemispheres were hierarchically clustered to depict the differences between normothermia (*n*=7) and hypothermia (*n*=8) treatments. The fold changes were expressed as log_2_ (test/sham control) values. Each metabolite or animal is represented by a single row or column of boxes. The *red* and *blue* texts denote standard amino acids and metabolites related to acetylation, respectively. * and ^†^*P*<0.05 for increases and decreases compared with the sham control, respectively.

To pinpoint specific metabolites and related metabolic pathways responding to hypothermia, we compared the levels of metabolites from brains after reoxygenation between animals subjected to normothermia (38°C) and those subjected to hypothermia (30°C). Since these animals underwent a 5.5-hour nonfeeding period, the comparison was made against sham control animals that were not subjected to H-I but that were separated from their dams for the same duration. A cluster analysis (Figure 4) showed that hypothermia did not induce homogeneous patterns of metabolic downregulation, suggesting that hypothermia does not result in a simple slowing of metabolism, but rather leads to coordinated effects on multiple regulatory processes.

**Figure 5:**
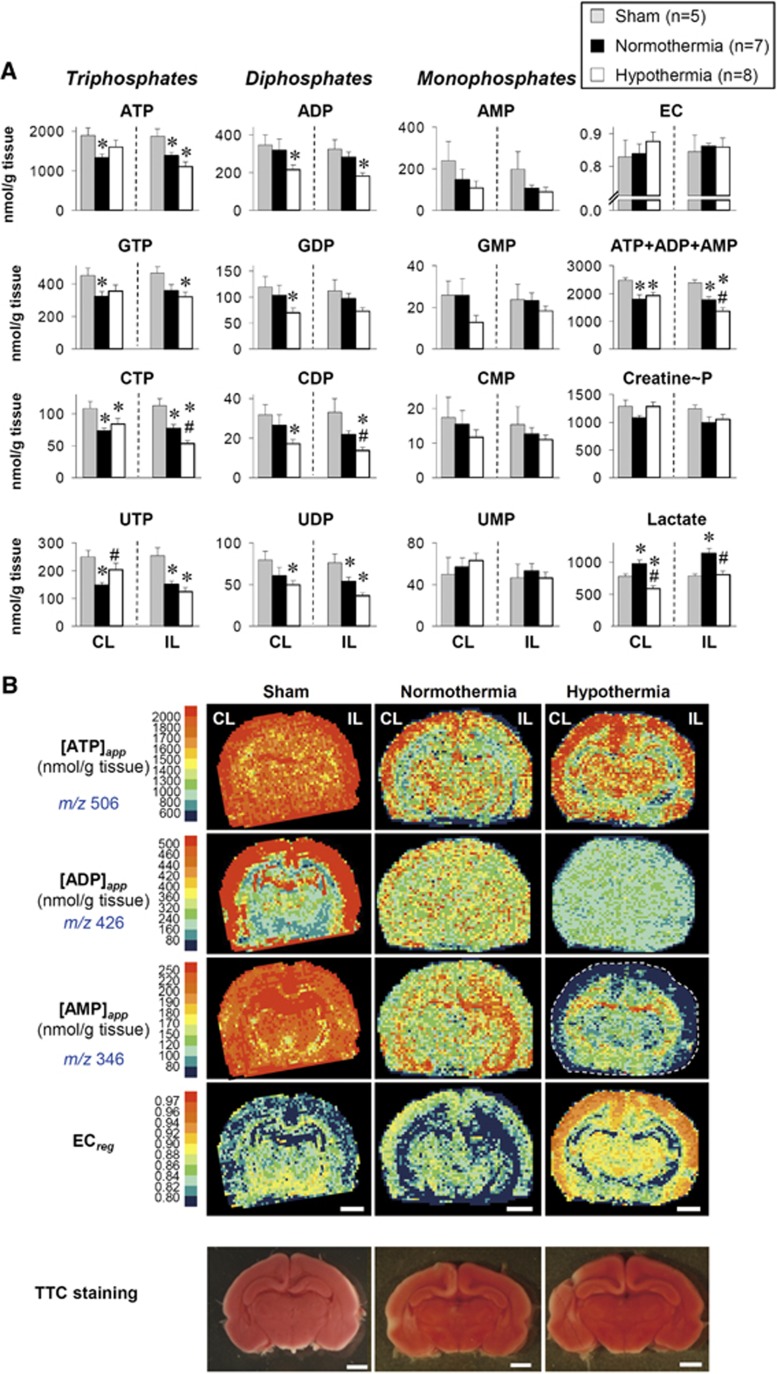
Energetic status at a 3-hour reoxygenation after the hypoxia-ischemia (H-I). (**A**) Alterations in high-energy phosphates and related metabolites in the contralateral (CL) and ipsilateral (IL) hemispheres. The tissue contents were determined by capillary electrophoresis-electrospray ionization mass spectrometry (CE/ESI/MS). Values are mean±s.e.m. * and ^#^*P*<0.05 compared with sham and normothermia, respectively. (**B**) Quantitative imaging mass spectrometry (Q-IMS) showing spatial distributions of apparent contents of adenylates ([ATP]_*app*_, [ADP]_*app*_, [AMP]_*app*_) and energy charge (EC). These maps for ATP, ADP, AMP, and EC were constructed on the same tissue. Hypothermia caused marked decreases in [AMP]_*app*_ especially in the cortex, leading to increases in EC. Reoxygenation with hypothermia treatment completely reversed the low energetic state induced by the H-I insult. (*Bottom*) Staining with 2,3,5-triphenyltetrazolium chloride (TTC) cannot detect apparent metabolic alterations occurring at 3 hours after H-I. Scale bars=2 mm.

Using the Rice-Vannucci model of neonatal H-I, in which a common carotid artery is unilaterally ligated, hypothermia was found to produce different metabolic responses between the CL and IL hemispheres (Figure 4). Hypothermia attenuated hypoxanthine and inosine (two purine-degraded metabolites) as well as lactate in both hemispheres (Cluster I_*CL*_ and I_*IL*_). However, the responses of many metabolites to hypothermia were not necessarily identical between the two hemispheres. For example, in the CL, cooling caused global decreases in the standard amino acids, except for branched-chain amino acids (Cluster III_*CL*_). Since brain ischemia in adult mice causes rapid protein degradation that coincided with a global elevation of amino acids,^10^ these results suggested that hypothermia prevents protein degradation. Conversely, in the IL, increases in many amino acids were observed (Cluster III_*IL*_), except for Ser, Glu, Gln, Asp, and Asn. Considering that Glu and Gln are mainly derived from circulating glucose,^25^ their deceases could be indicative of reduced blood flow to the IL under hypothermia. Cooling, however, did not cause an impaired energetic state. On the contrary, not only the CL tissues but also the IL tissues of hypothermia were metabolically active as judged by high energy charge (EC) (Figure 5). Indeed, heterogeneous distributions of EC values under hypothermia resemble those of the normal brain (see Figure 3B). Evident normalization of EC patterns appear to be effectively brought about by the marked decreases in values of [AMP]_*app*_ seen in the cortex regions under hypothermia.

### Hypothermia Diminishes Metabolites Containing an Acetyl Group in Both Contralateral and Ipsilateral Hemispheres

Metabolomics profiling showed that hypothermia diminishes carbon biomass related to acetyl groups, such as pyruvate and acetyl-CoA; conversely, it causes increases in deacetylated metabolites, such as carnitine and choline (Figure 4). Unlike the metabolic responses of energy metabolites and amino acids to cooling that differ between the two hemispheres, decreases in pyruvate and acetyl-CoA occurred commonly in both hemispheres. This trans-hemispheric commonality led us to closely examine how therapeutic hypothermia changes the dynamics of acetyl moiety-related metabolites.

**Figure 6:**
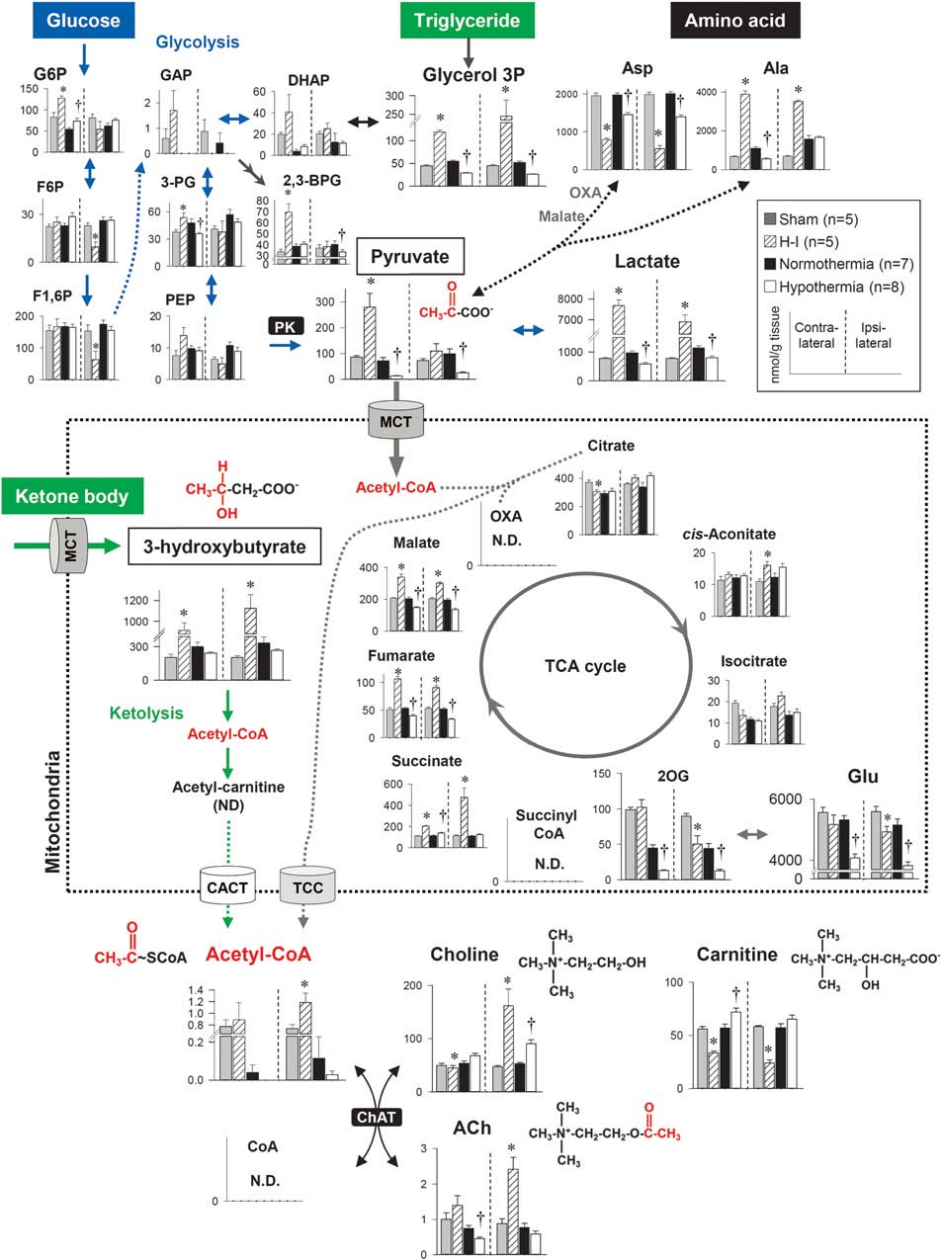
Contents of brain metabolites involved in central carbon metabolism in neonates. Differences in the brain contents of metabolites among sham, hypoxia-ischemia (H-I), normothermia, and hypothermia groups are superimposed on metabolic pathways including glycolysis (*blue arrows*), ketolysis (*green arrows*), amino-acid metabolism, and acetylcholine (ACh) synthesis. Hypothermia diminished metabolites containing an acetyl-group in both the contralateral and ipsilateral hemispheres. Data indicate the mean±s.e.m. of five to eight animals for each group. *P<0.05, *versus* sham. ^†^*P*<0.05, *versus* normothermia. N.D., not detected; CACT, carnitine/acetylcarnitine translocase; ChAT, choline acetyltransferase, MCT, monocarboxylate transporter; PK, pyruvate kinase; TCC, tricarboxylate carrier.

**Figure 7:**
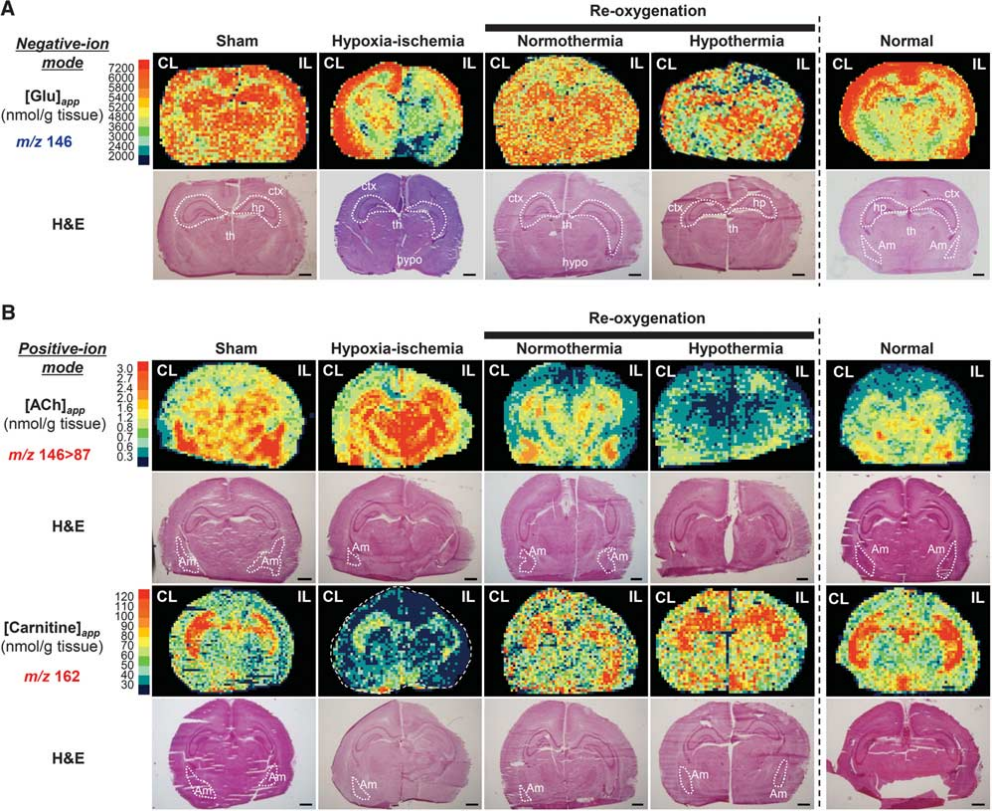
Quantitative imaging mass spectrometric (Q-IMS) visualization of the effects of therapeutic hypothermia on glutamate, acetylcholine (ACh), and carnitine contents. (**A**) Negative-ion mode was used to construct the content maps of glutamate. In the sham group, apparent contents of glutamate ([Glu]_*app*_) were high in cortex (*ctx*), hippocampus (*hp*) and thalamus (*th*). Under normothermia, the spatial distribution of glutamate became rather homogeneous. Hypothermia appeared to reverse such a pattern to the one seen in the normal group by reducing [Glu] in certain areas such as hypothalamus (*hypo*). (**B**) Positive-ion mode was used to construct the content maps of ACh and carnitine. MS/MS imaging of ACh where the ion transition signature of *m/z* 146>87 was used for the mapping (*top*). In the sham group, the [ACh]_*app*_ values were high in the amygdala (*Am*) and the hippocampus, but relatively low in the cortex. Hypoxia-ischemia (H-I) caused substantial increases in [ACh]_*app*_ in hippocampus and thalamus. Subsequently, a 3-hour reoxygenation under both normothermic and hypothermic conditions substantially decreased [ACh]_*app*_. Note that [ACh]_*app*_ after normothermic treatment was lower than its pre-H-I values in many regions. Content maps for carnitine (*bottom*). The [carnitine]_*app*_ values were high in the hippocampus in the control but were substantially decreased by the H-I. Reoxygenation resulted in the return of hippocampal carnitine levels to their preinsult levels, with the enhancement being greater in the hypothermia group than in the normothermia group. The dynamics for carnitine appeared to be opposite to that for ACh. A map consists of ~40 × ~60 rectangles, each defining an area of 0.2 × 0.2 mm^2^. Scale bar=1 mm. Each Q-IMS image is accompanied by hematoxylin & eosin (H&E) staining of the very same section.

Suckling rats are ketogenic because of the high fat content of rodent milk, and ketone bodies can provide up to 60% of the cerebral energetic fuel.^26^ In the brains of 7-day postnatal rats, pyruvate and 3-hydroxybutyrate are the two major precursors that donate acetyl groups to form acetyl-CoA, a key metabolite residing at the metabolic crossroads of glycolysis, ketolysis, and amino-acid metabolism. Figure 6 shows the contents of metabolites in these critical pathways. First, in glycolysis, hypothermia dramatically attenuated the conversion of phosphoenol pyruvate to pyruvate, suggesting that pyruvate kinase is sensitive to cooling. The decrease in lactate production in response to hypothermia led us to conclude that cooling suppresses glycolysis. Second, hypothermia decreased alanine, aspartate, and glutamate, three types of amino acids that are present in large amounts (Supplementary Figure S1). As shown in the Q-IMS analyses (Figure 7A), the spatial distribution of glutamate in the normothermia was rather homogeneous; *i.e.*, levels of glutamate were elevated over whole regions of the brain. By contrast, in the hypothermia, it became heterogeneous where cortex and hippocampus displayed higher contents of glutamate than other regions. Such structural heterogeneity appears to resemble that of the normal brain, suggesting that hypothermia contributes to normalizing spatial distributions of the excitatory neurotransmitter. Finally, in lipid metabolism, hypothermia caused decreases in glycerol 3-phosphate and 3-hydroxybutyrate, which can be converted to acetyl-CoA *via* the ketolytic pathway in mitochondria. These observations led us to hypothesize that hypothermia attenuates ACh synthesis *via* the coordinated suppression of acetyl-CoA contents, which in turn mediates neuroprotection.

### Hypothermia Diminishes Acetylcholine with a Reciprocal Increase in Carnitine

In the peripheral nervous system, the well-documented role of ACh is to act as a fast-acting neurotransmitter at neuromuscular junctions. In the central nervous system, however, it coordinates the firing of multiple groups of neurons by changing the presynaptic release of neurotransmitters and neuronal excitability;^27^ thus, it modulates many central nervous system functions. The ACh signaling has been suggested to play roles in stress responses. Therefore, we examined the spatial distributions of ACh contents in the brain and how different conditions change them using Q-IMS technology, which permits quantitative intergroup comparisons.^10, 13^

To determine the distribution of ACh in brain sections, we conducted MS/MS imaging.^25^ Through an ion transition from *m/z* 146 to *m/z* 87, ACh was successfully visualized using a high S/N ratio (Supplementary Figure S2). Figure 7B shows the content maps for ACh, where apparent area-specific ACh contents ([ACh]_*app*_) were assigned in units of nmol/g tissue for four different groups. [ACh]_*app*_ in the amygdala exhibited the highest value, while that in the cortex was rather low in the sham control. The H-I insult caused a noticeable increase in [ACh]_*app*_ in the hippocampus and thalamus. Subsequently, 3 hours of reoxygenation under both normothermic and hypothermic conditions reversed [ACh]_*app*_ to below the pre-H-I values. The extent of the reduction was greater under the hypothermic conditions than under the normothermic conditions.

Such region-specific changes in the ACh content led us to investigate changes in the patterns of deacetylated metabolites involved in ACh synthesis. Among several metabolites, we were able to construct content maps for carnitine. To identify an ion peak at *m/z* 162.11, we conducted MS/MS analysis comparing fragment fingerprints acquired from the tissue and standard compounds. A perfect match of these spectra assigns these metabolites to be carnitine (Supplementary Figure S2). To our knowledge, this is the first study to show the spatial distribution of carnitine. In the sham control, the values of [carnitine]_*app*_ in the hippocampus were high, but these values decreased substantially after the H-I insult. Reoxygenation resulted in reversal of the hippocampal carnitine levels to their preinsult levels. The extent of such an increase was greater in the hypothermic group than in the normothermic group. The greater the amount of carnitine, the lower the ACh level in the hippocampus and *vice versa*; *i.e.,* an inverse correlation was observed between the ACh and carnitine contents in the same region. In summary, the patterns of ACh and carnitine complemented each other, forming a metabolically delineated boundary around the hippocampus, which is the center of learning and memory.

## Discussion

In the present study, we provided evidence that therapeutic hypothermia mediates neuroprotection *via* the coordinated suppression of the acetyl-CoA content, which in turn downregulates the production of ACh in specific regions of the brain. We substantiated these findings using a two-step approach. First, using a nontargeted metabolome analysis to identify metabolic pathways that responded to cooling, we found that multiple metabolic pathways giving rise to acetyl-CoA, such as glycolysis and ketolysis, responded to a lowering of the temperature. Subsequently, with Q-IMS, we showed that hypothermia causes a reduction in the ACh content with a concurrent rise in the carnitine content in the hippocampus.

From the viewpoint of improving treatment for neonatal H-I, the most important feature of the current findings is that the decreased ACh content was linked to a better neurologic outcome. This downregulation of ACh production may explain why therapeutic hypothermia can achieve neuroprotection. It is worth noting that the hypothermic treatment did not lower the ACh content when it was conducted on the healthy neonates without H-I (data not shown). Pace-Schott and Hobson^28^ claimed that ‘ACh is a neurotransmitter of the activated brain'. This proposal was based on cumulative findings that ACh concentrations in the hippocampus^29^ and the thalamus^30^ are higher during REM (rapid eye movement) sleep and wakefulness than during non-REM sleep, suggesting that a state-dependent release of ACh mediates the periodicity of the sleep-wake cycle. A recent study suggesting a correlation between the delayed onset of sleep-wake cycling and a favorable outcome in hypothermic-treated infants with encephalopathy^31^ may provide a link between the above proposal and the clinical relevance of neuroplasticity. It could, therefore, be argued that the suppressed production of ACh may cause the delayed onset of waking, resulting in a neuroprotective effect. However, the mechanisms by which this delay mediates neuroprotection are ambiguous at present. We speculated that the quiescence-like state created by hypothermia is not a simple preservation of metabolic stores or a rendering of the tissue in a state of 'suspended animation'.^32^ On the contrary, this state appears to be a metabolic-decision-making period to optimize the fates of neurons and glial cells when confronted with limited biosynthetic precursors and energy fuels. Such a situation is supported by our metabolome analysis, which showed that hypothermia causes an increase in phosphoribosyl pyrophosphate (Figure 4), the building block of nucleic acids. Such an idea deserves further examination.

Although there are multiple mechanisms explaining how hypothermia downregulates the production of ACh, a decrease in the acetyl-CoA pool that donates acetyl moieties to choline to form ACh is a common theme. Two major sources of acetyl groups are pyruvate and ketone bodies, both of which are decreased by hypothermia (Figure 6). First, pyruvate is provided by glucose as the end product of glycolysis. The metabolic responsiveness to hypothermia becomes apparent at the conversion between phosphoenol pyruvate to pyruvate, suggesting that the reaction catalyzed by pyruvate kinase is a target of cooling. Another possibility is supported by previous studies performed *in vitro* that show a temperature dependency of pyruvate kinase.^33^ Second, pyruvate can also be produced *via* transamination reactions of amino acids, such as aspartate and alanine. Third, considering the exceedingly high fraction of lipids contained in mothers' milk, which corresponds to approximately 60% (w/v),^34^ acetoacetate and 3-hydroxybutyrate derived from ketogenesis in the liver are likely to be at least as important as glucose as a fuel during this stage of development.^35^ In this model, ketone bodies can cross the blood–brain barrier by means of monocarboxylate transport systems,^36^ which occurs at a higher rate in suckling mice than in the adults.^37^ Our metabolome approach clearly showed that hypothermia suppresses the brain tissue contents of acetyl-CoA, which resides at the metabolic crossroad of glycolysis, amino-acid catabolism, and ketolysis, in a coordinated manner.

Our strategy using Q-IMS enabled intergroup comparisons of regional ACh and carnitine contents across all the different experimental conditions (Figure 7). The different patterns induced by different treatments showed a clear reciprocal relation between the ACh and carnitine contents, especially in the hippocampus and to a lesser extent in the amygdala. This inverse correlation suggests a role of endogenous carnitine in the modulation of neuroplasticity through its effects on ACh synthesis in specific regions of the brain. What might be the mechanisms whereby this inverse relation is brought about? The synthesis of ACh is catalyzed by choline acetyltransferase (ChAT), the enzyme that transfers the acetyl moiety from acetyl-CoA to choline, producing ACh and CoA. Here, the structural similarity between carnitine and choline has led previous investigators to examine the substrate specificity of ChAT *in vitro*. Studies using purified ChAT have shown a strong preference for choline over carnitine as an acetyl acceptor.^38, 39^ These results minimize the possibility that carnitine competes with choline for ChAT, thereby inhibiting its activity. In contrast, a study using cultured cortical neurons isolated from suckling rats showed that the addition of exogenous carnitine inhibits ACh synthesis by 30%.^40^ Moreover, various studies have suggested that the exogenous administration of carnitine reduces brain injury upon H-I.^41^ Such observations further reinforce the significance of the anatomically specific regulation of ACh and carnitine metabolism.

One possibility to explain the neuroprotective effect brought about by hypothermia is the decrease in glutamate contents. H-I causes accumulation of extracellular glutamate leading to disturbance of synaptic function known as ‘excitatory toxicity'.^42^ Our results indicate that hypothermia not only attenuates glutamate synthesis but also normalize spatial distributions of this major excitatory neurotransmitter. Although our approach cannot separate extracellular events from those of intracellular events, decreases in glutamate synthesis can be beneficial. The possibility of this kind deserves further investigation.

In summary, our study suggests that lowering the ACh level in specific regions of the brain comprises part of the multifaceted mechanisms by which therapeutic hypothermia achieves neuroprotection. As the regulations of other potentially protective metabolic alterations are also likely to occur during hypothermia, our Q-IMS analyses should be further exploited to help design more effective therapeutic strategies for H-I injury.
