# Supplementary material for: Therapeutic hypothermia achieves neuroprotection via a decrease in acetylcholine with a concurrent increase in carnitine in the neonatal hypoxia-ischemia
Source: J Cereb Blood Flow Metab. 2015 Jan 14;35(5):794–805. doi: 10.1038/jcbfm.2014.253 (PMC4420853; doi:10.1038/jcbfm.2014.253)
Supplement: Supplementary Information [file jcbfm2014253x1.pdf]

# Supplementary Information

# Therapeutic hypothermia achieves neuroprotection *via* a decrease in acetylcholine with a concurrent increase in carnitine in the neonatal hypoxia-ischemia

Toshiki Takenouchi[1,2†], Yuki Sugiura[1,3†], Takayuki Morikawa[1,4†], Tsuyoshi Nakanishi[1,5], Yoshiko Nagahata[1,4], Tadao Sugioka[1], Kurara Honda[1,3], Akiko Kubo[1,4], Takako Hishiki[1,4], Tomomi Matsuura[1,4], Takao Hoshino[1], Takao Takahashi[2], Makoto Suematsu[1,4*], Mayumi Kajimura[1,4*]

[1]Department of Biochemistry, Keio University School of Medicine, Tokyo 160-8582
[2]Department of Pediatrics, Keio University School of Medicine, Tokyo 160-8582
[3]JST Precursory Research for Embryonic Science and Technology (PRESTO) Project,
[4]JST Exploratory Research for Advanced Technology (ERATO) Suematsu Gas Biology Project, Tokyo 160-8582, Japan
[5]MS Business Unit, Shimadzu Corporation, Kyoto 604-8511, Japan

† These authors contributed equally.

Running Head (50 characters not including space): **Hypothermia attenuates ACh synthesis**

**[*]Address for Correspondence:**
Mayumi Kajimura (myk30@z5.keio.jp)
Makoto Suematsu (gasbiology@z6.keio.jp)
Department of Biochemistry
Keio University School of Medicine,
35 Shinanomachi, Shinjuku-ku
Tokyo 160-8582, Japan
Phone: +81-3-5363-3461
Fax: +81-3-5363-3466

***One table and two figures are included as the Supplemental Information.***

## Supplementary Table 1. *Comparative values for metabolites' contents quantified by CE/ESI/MS analyses in the normal, sham, hypoxia-ischemia, normothermia and hypothermia groups*



| | Contralateral | | | | | Ipsilateral | | | | |
|---|---|---|---|---|---|---|---|---|---|---|
| | **Normal (5)** | **Sham (5)** | **Hypoxia-Ischemia (5)** | **Normothermia (7)** | **Hypothermia (8)** | **Normal (5)** | **Sham (5)** | **Hypoxia-Ischemia (5)** | **Normothermia (7)** | **Hypothermia (8)** |
| **Glycolysis** | | | | | | | | | | |
| Glucose 1-phosphate | 36.6 ± 4.8 | 21.3 ± 1.1 * | 23.5 ± 2.2 | 29.0 ± 2.3 | 28.6 ± 1.4 | 41.4 ± 5.8 | 21.6 ± 0.8 * | 14.9 ± 3.4 | 32.8 ± 3.2 | 27.3 ± 1.1 |
| Glucose 6-phosphate | 67.5 ± 5.4 | 82.9 ± 10.5 | 127 ± 5.1 † | 54.0 ± 4.2 | 73.6 ± 7.1 # | 70.2 ± 4.3 | 80.9 ± 8.6 | 53.6 ± 19.0 | 61.7 ± 7.5 | 75.4 ± 4.2 |
| Fructose 6-phosphate | 19.6 ± 1.6 | 22.4 ± 1.7 | 25.4 ± 3.2 | 23.0 ± 1.7 | 28.9 ± 2.3 | 21.0 ± 2.1 | 23.0 ± 1.8 | 9.7 ± 3.2 † § | 26.2 ± 2.4 | 26.4 ± 2.2 |
| Fructose 1,6-bisphosphate | 155 ± 19.3 | 154 ± 17.9 | 167 ± 24.2 | 168 ± 12.6 | 165 ± 9.6 | 148 ± 10.7 | 154 ± 18.7 | 63.2 ± 26.9 † § | 174 ± 12.2 | 156 ± 12.8 |
| Dihydroxyacetonephosphate | 19.9 ± 2.3 | 19.8 ± 2.1 | 41.1 ± 16.2 | 3.9 ± 1.9 | 8.4 ± 1.9 | 29.1 ± 2.1 | 20.6 ± 2.4 * | 25.1 ± 5.3 | 12.4 ± 8.6 | 11.6 ± 1.9 |
| 2,3-Bisphosphoglycerate | 31.7 ± 2.2 | 33.7 ± 2.2 | 69.5 ± 7.6 † | 38.6 ± 2.4 | 40.3 ± 2.5 | 36.2 ± 3.8 | 36.8 ± 3.3 | 38.4 ± 4.6 § | 40.2 ± 3.4 | 33.7 ± 1.9 |
| 3-Phosphoglycerate | 44.9 ± 4.6 | 37.6 ± 2.8 | 53.7 ± 4.7 † | 47.8 ± 4.6 | 35.9 ± 1.7 # | 55.1 ± 5.7 | 41.0 ± 3.4 | 37.8 ± 12.2 | 56.7 ± 6.0 | 48.7 ± 3.6 |
| Phosphoenolpyruvate | 9.4 ± 1.4 | 7.7 ± 1.6 | 13.9 ± 2.5 | 9.8 ± 1.0 | 9.1 ± 1.2 | 11.7 ± 0.9 | 6.4 ± 0.7 * | 5.0 ± 2.0 § | 10.8 ± 1.2 | 8.9 ± 1.4 |
| Pyruvate | 84.8 ± 5.1 | 87.3 ± 5.3 | 281 ± 53.5 † | 72.7 ± 12.7 | 13.2 ± 2.5 # | 86.5 ± 5.8 | 74.1 ± 6.5 | 110 ± 29.6 § | 99.9 ± 19.3 | 25.5 ± 4.3 # |
| Lactate | 793 ± 35.7 | 781 ± 34.9 | 7681 ± 277 † | 978 ± 60.9 | 590 ± 42.2 # | 806 ± 40.8 | 785 ± 30.9 | 6897 ± 336 † | 1139 ± 77.4 | 806 ± 54.2 # |
| **Glycerol phosphate shuttle** | | | | | | | | | | |
| Glycerol 3-phosphate | 47.3 ± 1.4 | 45.1 ± 1.9 | 119 ± 4.4 † | 54.8 ± 2.9 | 29.1 ± 1.2 # | 47.7 ± 2.0 | 45.6 ± 2.2 | 245 ± 43.5 † § | 52.3 ± 2.6 | 26.6 ± 1.3 # |
| **TCA Cycle** | | | | | | | | | | |
| Acetyl CoA | 0.7 ± 0.2 | 0.8 ± 0.2 | 0.9 ± 0.3 | 0.1 ± 0.1 | N.D. | 0.6 ± 0.1 | 0.7 ± 0.1 | 1.2 ± 0.2 † | 0.2 ± 0.2 | 0.1 ± 0.1 |
| Citrate | 360 ± 13.6 | 372 ± 13.9 | 305 ± 13.9 † | 291 ± 16.6 | 306 ± 21.3 | 366 ± 10.9 | 359 ± 7.6 | 402 ± 19.9 § | 337 ± 31.2 | 417 ± 21.5 |
| cis-Aconitate | 10.8 ± 0.3 | 11.5 ± 1.1 | 13.2 ± 0.8 | 12.2 ± 1.1 | 12.8 ± 0.7 | 10.7 ± 0.7 | 11.0 ± 0.8 | 16.1 ± 1.3 † | 12.3 ± 1.4 | 15.5 ± 1.3 |
| Isocitrate | 15.8 ± 1.5 | 19.4 ± 1.3 | 13.6 ± 2.5 | 11.7 ± 1.0 | 10.9 ± 0.7 | 15.0 ± 0.9 | 17.8 ± 1.5 | 22.8 ± 1.8 § | 13.7 ± 1.8 | 15.0 ± 1.8 |
| 2-Oxoglutarate | 67.1 ± 3.9 | 98.8 ± 3.8 * | 102 ± 10.8 | 44.5 ± 5.0 | 12.8 ± 1.4 # | 60.7 ± 4.1 | 89.7 ± 4.0 * | 50.4 ± 11.5 † § | 44.2 ± 7.1 | 12.7 ± 2.2 # |
| Succinate | 86.3 ± 2.3 | 112 ± 2.0 * | 204 ± 5.4 † | 114 ± 4.8 | 139 ± 5.7 # | 85.3 ± 2.7 | 115 ± 3.7 * | 476 ± 87.1 † § | 110 ± 6.2 | 125 ± 6.2 |
| Fumarate | 49.4 ± 2.8 | 51.5 ± 3.4 | 107 ± 4.8 † | 53.2 ± 1.8 | 40.2 ± 2.5 # | 57.0 ± 2.0 | 53.2 ± 2.9 | 90.8 ± 4.4 † § | 52.3 ± 2.6 | 33.6 ± 1.4 # |
| Malate | 202 ± 6.2 | 207 ± 3.7 | 340 ± 19.7 † | 204 ± 11.3 | 149 ± 5.1 # | 187 ± 5.7 | 204 ± 8.3 | 302 ± 9.0 † | 195 ± 10.8 | 137 ± 7.4 # |
| **Pentose phosphate pathway** | | | | | | | | | | |
| 6-Phosphogluconate | 13.4 ± 1.0 | 16.5 ± 1.1 | 13.7 ± 3.1 | 15.3 ± 1.1 | 12.0 ± 0.6 # | 13.8 ± 0.9 | 15.4 ± 1.3 | 5.6 ± 1.7 | 13.3 ± 0.8 | 10.8 ± 1.0 |
| Ribulose 5-phosphate | 13.6 ± 0.5 | 17.0 ± 0.7 * | 16.9 ± 4.7 | 7.8 ± 0.8 | 7.4 ± 0.7 | 14.7 ± 0.5 | 16.7 ± 1.1 | 13.9 ± 1.1 | 8.9 ± 2.1 | 8.9 ± 0.6 |
| Ribose 5-phosphate | 4.5 ± 0.3 | 6.0 ± 1.1 | 6.9 ± 2.3 | 2.0 ± 0.7 | 2.5 ± 0.5 | 5.5 ± 0.5 | 5.5 ± 0.6 | 4.6 ± 0.7 | 2.5 ± 1.1 | 3.9 ± 0.5 |
| D-Sedoheptulose 7-phosphate | 35.9 ± 2.8 | 42.6 ± 2 | 43.2 ± 3.4 | 45.3 ± 4.1 | 53.7 ± 2.4 | 31.2 ± 3.3 | 41.2 ± 1.3 * | 18.9 ± 5.1 † § | 47.9 ± 3.5 | 45.2 ± 3.1 |
| **Nucleic acid synthesis** | | | | | | | | | | |
| PRPP | 7.6 ± 0.8 | 7.0 ± 0.6 | 9.3 ± 0.9 | 5.0 ± 0.4 | 7.7 ± 0.9 # | 7.5 ± 0.3 | 6.7 ± 0.4 | 8.5 ± 0.5 † | 12.9 ± 3.5 | 30.4 ± 1.6 # |

| | Contralateral | | | | | Ipsilateral | | | | |
|---|---|---|---|---|---|---|---|---|---|---|
| | Normal (5) | Sham (5) | Hypoxia-Ischemia (5) | Normothermia (7) | Hypothermia (8) | Normal (5) | Sham (5) | Hypoxia-Ischemia (5) | Normothermia (7) | Hypothermia (8) |
| **Methylated products** | | | | | | | | | | |
| Met | 34.8 ± 1.4 | 18.5 ± 2.4 * | 19.8 ± 6.2 | 17.2 ± 2.0 | 11.1 ± 2.0 # | 36.2 ± 1.9 | 16.4 ± 1.2 * | 49.5 ± 13.8 | 27.1 ± 5.0 | 31.5 ± 5.0 |
| SAM | 32.3 ± 1.6 | 31.7 ± 1.3 | 33.1 ± 1.2 | 26.1 ± 0.9 | 22.2 ± 1.6 | 33.4 ± 1.7 | 32.0 ± 1.1 | 37.6 ± 0.8 † § | 31.7 ± 1.9 | 27.2 ± 1.6 |
| Spermidine | 32.0 ± 1.7 | 32.1 ± 1.3 | 35.0 ± 2.2 | 35.8 ± 1.5 | 35.2 ± 1.0 | 33.3 ± 1.1 | 31.2 ± 0.7 | 32.6 ± 1.1 | 36.4 ± 1.1 | 31.6 ± 1.6 # |
| Spermine | 13.5 ± 1.2 | 11.6 ± 0.8 | 14.2 ± 1.4 | 14.4 ± 1.2 | 15.4 ± 1.2 | 15.7 ± 1.3 | 11.6 ± 1.0 * | 9.1 ± 2.3 | 14.1 ± 1.1 | 11.7 ± 1.2 |
| SAH | 0.9 ± 0.1 | 0.7 ± 0.2 | 0.8 ± 0.2 | 1.0 ± 0.1 | 1.0 ± 0.1 | 0.6 ± 0.1 | 0.6 ± 0.1 | 1.8 ± 0.4 † | 1.0 ± 0.1 | 1.1 ± 0.1 |
| Cystathionine | 87.0 ± 4.2 | 73.7 ± 10.2 | 116 ± 9.0 † | 97.7 ± 11.6 | 97.8 ± 12.3 | 85.3 ± 5.6 | 72.9 ± 11.4 | 112 ± 8.0 † | 120 ± 19.4 | 123 ± 10.3 |
| Hypotaurine | 180 ± 9.0 | 142 ± 14.7 | 254 ± 26.9 † | 184 ± 14.2 | 171 ± 19.3 | 196 ± 9.7 | 134 ± 10.9 * | 231 ± 20.1 † | 204 ± 15.7 | 186 ± 15.1 |
| Taurine | 9298 ± 376 | 9781 ± 279 | 10495 ± 428 | 11253 ± 250 | 10793 ± 528 | 9307 ± 153 | 9548 ± 371 | 9329 ± 262 | 10943 ± 476 | 9727 ± 363 |
| Thiotaurine | 22.9 ± 1.0 | 25.9 ± 1.6 | 28.4 ± 1.3 | 28.5 ± 0.9 | 28.9 ± 1.8 | 23.0 ± 0.6 | 23.7 ± 0.9 | 24.5 ± 1.5 | 26.4 ± 2.3 | 24.5 ± 2.1 |
| GSH | 156 ± 59.2 | 24.9 ± 18.2 | 56.4 ± 28.3 | 7.8 ± 1.0 | 5.0 ± 0.3 # | 77.7 ± 26.7 | 6.8 ± 0.9 | 165 ± 59.9 | 21.2 ± 14.7 | 5.2 ± 0.4 |
| GSSG | 667 ± 24.8 | 684 ± 13.2 | 731 ± 68.2 | 712 ± 19.2 | 720 ± 32.2 | 752 ± 17.5 | 663 ± 16.2 * | 548 ± 70.9 | 694 ± 44.0 | 558 ± 36.2 # |
| N-Methyl-Arg | 0.5 ± 0.2 | 0.3 ± 0.1 | 0.5 ± 0.1 | 0.5 ± 0.1 | 0.4 ± 0.1 | 0.3 ± 0.1 | 0.3 ± 0.1 | 0.4 ± 0.1 | 0.6 ± 0.1 | 0.6 ± 0.1 |
| ADMA | 1.5 ± 0.2 | 1.9 ± 0.2 | 2.8 ± 0.2 † | 2.1 ± 0.2 | 1.8 ± 0.2 | 1.7 ± 0.1 | 1.9 ± 0.2 | 2.3 ± 0.3 | 2.5 ± 0.2 | 2.6 ± 0.2 |
| SDMA | 0.9 ± 0.1 | 1.2 ± 0.1 * | 1.8 ± 0.2 † | 2.1 ± 0.2 | 1.4 ± 0.2 # | 0.9 ± 0.1 | 1.2 ± 0.1 * | 2.1 ± 0.2 † | 2.9 ± 0.3 | 2.6 ± 0.2 |
| **Urea cycle** | | | | | | | | | | |
| Ornithine | 44.7 ± 3.4 | 47.8 ± 2.5 | 49.5 ± 2.2 | 58.1 ± 3.7 | 63.5 ± 5.2 | 46.0 ± 1.4 | 48.2 ± 1.8 | 70.8 ± 7.7 † | 77.5 ± 6.8 | 117 ± 6.4 # |
| Citrulline | 139 ± 8.0 | 158 ± 9.0 | 187 ± 6.8 † | 195 ± 15.9 | 165 ± 11.4 | 149 ± 1.1 | 164 ± 6.1 | 206 ± 7.3 † | 231 ± 19.5 | 216 ± 13.3 |
| Creatine | 1491 ± 36.3 | 1539 ± 45 | 1949 ± 67.5 † | 1927 ± 56.0 | 1709 ± 55.5 # | 1598 ± 63.6 | 1518 ± 93.2 | 1860 ± 62.2 † | 1852 ± 74.2 | 1443 ± 55.4 # |
| Creatine phosphate | 1153 ± 78.7 | 1285 ± 116 | 764 ± 91.3 † | 1081 ± 45.0 | 1284 ± 76.8 # | 999 ± 54.6 | 1242 ± 69.4 * | 183 ± 100 † § | 991 ± 105 | 1054 ± 87.7 |
| Creatinine | 10.7 ± 0.4 | 13.4 ± 0.4 * | 15.7 ± 2.1 | 22.0 ± 1.5 | 17.1 ± 0.9 # | 11.0 ± 0.4 | 13.0 ± 0.6* | 14.2 ± 1.0 | 19.9 ± 1.7 | 16.3 ± 0.9 |
| Hydroxyproline | 93.4 ± 24 | 144 ± 36.9 | 162 ± 2.4 | 185 ± 9.9 | 48.9 ± 25.9 # | 117 ± 8.3 | 168 ± 9.9* | 152 ± 5.3 | 215 ± 9.6 | 60.9 ± 32.1 # |
| **Amino acids** | | | | | | | | | | |
| Gly | 897 ± 87.5 | 1227 ± 97.2 * | 1240 ± 72.0 | 1059 ± 86.7 | 999 ± 83.2 | 983 ± 65.6 | 1251 ± 69.4 * | 1709 ± 243 | 1371 ± 151 | 1526 ± 51.2 |
| Ala | 618 ± 39.7 | 683 ± 28.1 | 3880 ± 177 † | 1094 ± 94.8 | 569 ± 52.9 # | 675 ± 35.8 | 702 ± 18.3 | 3517 ± 67.8 † | 1564 ± 209 | 1661 ± 69.8 |
| Ser | 696 ± 22.5 | 864 ± 23.4 * | 729 ± 52.0 | 862 ± 47.0 | 649 ± 25.1 # | 760 ± 23.2 | 897 ± 42.1 * | 840 ± 52.2 | 1115 ± 112 | 1040 ± 43.5 |
| Thr | 428 ± 19.9 | 402 ± 7.3 | 680 ± 85.1 † | 599 ± 49.2 | 476 ± 35.1 | 458 ± 18.2 | 415 ± 32.1 | 754 ± 95.7 † | 849 ± 104 | 775 ± 56.9 |
| Val | 128 ± 14.0 | 115 ± 12.8 | 178 ± 9.3 † | 186 ± 11.9 | 244 ± 18.8 # | 134 ± 9.4 | 114 ± 5.1 | 409 ± 66.2 † § | 271 ± 35.1 | 461 ± 23.5 # |
| Ile | 42.8 ± 6.4 | 46.1 ± 5.3 | 91.2 ± 6.3 † | 76.8 ± 7.4 | 115 ± 8.5 # | 49.6 ± 4.9 | 46.7 ± 2.2 | 211 ± 36.4 † § | 107 ± 15.6 | 211 ± 11.5 # |
| Leu | 124 ± 13.2 | 132 ± 10.4 | 158 ± 10.1 † | 202 ± 14.1 | 262 ± 19.5 # | 123 ± 9.1 | 131 ± 6.1 | 357 ± 58.4 † § | 282 ± 34.8 | 479 ± 18.5 # |
| Lys | 237 ± 24.3 | 228 ± 12.7 | 383 ± 16.3 | 241 ± 15.9 | 216 ± 17.4 | 273 ± 8.7 | 237 ± 11.1 * | 532 ± 47.2 † § | 369 ± 52.5 | 521 ± 24.1 # |
| Arg | 82.2 ± 7.2 | 80.1 ± 4.5 | 71.1 ± 3.9 | 81.4 ± 4.0 | 70.1 ± 3.8 | 89.3 ± 4.0 | 79.7 ± 4.8 | 98.8 ± 9.1 § | 104 ± 6.8 | 143 ± 6.9 # |
| His | 140 ± 6.9 | 100 ± 2.9 * | 122 ± 3.6 † | 185 ± 6.7 | 168 ± 8.0 | 147 ± 4.1 | 100 ± 4.8 * | 186 ± 17.8 † § | 255 ± 22.3 | 291 ± 12.9 |
| Tyr | 196 ± 10.6 | 209 ± 5.1 | 387 ± 13.1 † | 233 ± 10.5 | 279 ± 23.7 | 209 ± 7.8 | 211 ± 7.1 | 602 ± 59.6 † § | 325 ± 30.8 | 516 ± 36.2 # |
| Phe | 66.7 ± 3.6 | 47.9 ± 3.8 * | 109 ± 3.2 † | 78.0 ± 5.3 | 79.9 ± 5.8 | 70.1 ± 2.5 | 46.6 ± 1.9 * | 193 ± 21.5 † § | 118 ± 16.4 | 160 ± 11.2 |
| Trp | 6.2 ± 1.0 | 5.5 ± 0.7 | 18.4 ± 3.5 † | 15.7 ± 2.5 | 13.2 ± 2.6 | 10.1 ± 2.1 | 4.8 ± 0.4 | 20.6 ± 5.2 † | 15.7 ± 4.2 | 21.7 ± 3.7 |
| Pro | 280 ± 22.2 | 368 ± 18.4 * | 708 ± 38.7 † | 428 ± 41.4 | 328 ± 34.1 | 297 ± 17.2 | 364 ± 8.6 * | 761 ± 37.7 † | 632 ± 85.6 | 697 ± 28.7 |
| Gln | 2777 ± 41.4 | 2958 ± 151 | 3212 ± 203 | 3531 ± 109 | 3136 ± 110 # | 3004 ± 61.6 | 3070 ± 75.7 | 1996 ± 400 † § | 3893 ± 77.5 | 2793 ± 110 # |
| Glu | 5140 ± 203 | 5560 ± 180 | 5176 ± 301 | 5321 ± 144 | 4081 ± 130 # | 5312 ± 133 | 5592 ± 175 | 4929 ± 185 † | 5157 ± 198 | 3829 ± 125 # |
| Asn | 149 ± 8.5 | 202 ± 13.1 * | 183 ± 8.2 | 224 ± 13.8 | 120 ± 7.5 # | 163 ± 3.4 | 200 ± 4.1 * | 231 ± 17.3 | 309 ± 32.2 | 255 ± 10.5 |
| Asp | 1925 ± 107 | 1946 ± 73.5 | 791 ± 40.2 † | 1974 ± 48.7 | 1450 ± 49.7 # | 2039 ± 92.3 | 1980 ± 66.8 | 554 ± 79.7 † § | 2003 ± 53.1 | 1393 ± 50.0 # |

## Nucleic acids

| | Contralateral Normal (5) | Contralateral Sham (5) | Contralateral Hypoxia-Ischemia (5) | Contralateral Normothermia (7) | Contralateral Hypothermia (8) | Ipsilateral Normal (5) | Ipsilateral Sham (5) | Ipsilateral Hypoxia-Ischemia (5) | Ipsilateral Normothermia (7) | Ipsilateral Hypothermia (8) |
|---|---|---|---|---|---|---|---|---|---|---|
| Adenine | 1.1 ± 0.2 | 1.9 ± 0.3 * | 3.8 ± 1.2 | 2.5 ± 0.7 | 3.3 ± 0.4 | 1.2 ± 0.1 | 2.1 ± 0.1 * | 2.3 ± 0.7 | 2.7 ± 0.7 | 3.7 ± 0.4 |
| Cytosine | 0.4 ± 0.2 | 1.6 ± 0.2 * | 1.8 ± 0.3 | 1.6 ± 0.3 | 1.9 ± 0.4 | 0.2 ± 0.1 | 1.5 ± 0.3 * | 1.6 ± 0.3 | 1.7 ± 0.3 | 2.0 ± 0.5 |
| Uracil | 7.2 ± 0.5 | 8.2 ± 0.4 | 14.1 ± 0.7 † | 13.2 ± 1.2 | 12.1 ± 1.3 | 7.0 ± 0.3 | 7.2 ± 0.5 | 53.4 ± 11.3 † § | 12.9 ± 0.8 | 12.3 ± 1.4 |
| Adenosine | 4.8 ± 1.1 | 5.4 ± 2.3 | 2.7 ± 1.0 | 6.9 ± 2.5 | 3.8 ± 1.3 | 5.9 ± 0.5 | 4.5 ± 1.2 | 8.5 ± 1.6 § | 4.5 ± 1.2 | 3.6 ± 0.9 |
| Guanosine | 1.4 ± 0.7 | 1.6 ± 0.4 | 1.6 ± 0.6 | 2.8 ± 0.5 | 1.8 ± 0.5 | 1.6 ± 0.2 | 1.3 ± 0.3 | 8.3 ± 1.9 † § | 2.2 ± 0.2 | 2.1 ± 0.7 |
| Cytidine | 4.8 ± 0.3 | 4.3 ± 0.5 | 10.3 ± 0.7 † | 6.4 ± 0.6 | 6.0 ± 0.5 | 5.1 ± 0.5 | 4.3 ± 0.4 | 46.8 ± 9.5 † § | 5.2 ± 0.6 | 3.9 ± 0.4 |
| Uridine | 20.6 ± 1.2 | 23.0 ± 0.4 | 33.5 ± 1.7 † | 40.4 ± 1.8 | 34.9 ± 3.6 | 21.6 ± 0.7 | 23.2 ± 1.1 | 97.3 ± 18.0 † § | 39.6 ± 1.2 | 31.8 ± 3.2 |
| Inosine | 3.6 ± 0.6 | 3.6 ± 0.7 | 8.8 ± 3.2 | 6.7 ± 1.0 | 4.2 ± 0.7 | 4.2 ± 1.1 | 3.4 ± 0.3 | 64.5 ± 16.4 † § | 3.6 ± 1.1 | 0.7 ± 0.5 # |
| AMP | 188 ± 77.5 | 239 ± 92.6 | 223 ± 54.3 | 148 ± 51.0 | 109 ± 33.1 | 114 ± 31.7 | 198 ± 84.5 | 441 ± 73.8 | 106 ± 15.3 | 90.3 ± 22.1 |
| GMP | 23.1 ± 6.3 | 26.0 ± 6.8 | 42.6 ± 13.0 | 25.9 ± 8.1 | 12.9 ± 3.5 | 24.3 ± 4.1 | 23.9 ± 7.4 | 145 ± 27.6 † § | 23.3 ± 3.9 | 18.5 ± 2.4 |
| CMP | 16.5 ± 4.9 | 17.4 ± 6.0 | 16.0 ± 3.8 | 15.4 ± 4.0 | 11.7 ± 2.2 | 15.1 ± 3.2 | 15.4 ± 5.1 | 12.8 ± 1.3 | 12.7 ± 1.8 | 11.0 ± 1.4 |
| TMP | 0.9 ± 0.2 | 0.8 ± 0.7 | 0.2 ± 0.2 | 0.6 ± 0.2 | 0.1 ± 0.1 # | 0.7 ± 0.2 | 0.1 ± 0.2 | N.D. | 0.2 ± 0.2 | 0.1 ± 0.1 |
| UMP | 55.0 ± 13.7 | 50.1 ± 16.1 | 50.0 ± 9.7 | 56.9 ± 9.1 | 63.4 ± 7.0 | 52.8 ± 9.7 | 46.6 ± 13.1 | 42.9 ± 3.0 | 53.3 ± 7.3 | 46.4 ± 5.6 |
| IMP | 29.3 ± 8.7 | 32.9 ± 11.3 | 46.0 ± 16.9 | 29.1 ± 7.2 | 18.9 ± 4.1 | 20.2 ± 4.4 | 30.0 ± 11.0 | 83.2 ± 10.9 † | 23.6 ± 3.4 | 18.6 ± 3.0 |
| cAMP | 0.7 ± 0.3 | 0.7 ± 0.4 | 0.2 ± 0.2 | 0.5 ± 0.2 | 0.5 ± 0.2 | 0.6 ± 0.3 | 0.8 ± 0.2 | 0.3 ± 0.2 † | 0.7 ± 0.1 | 0.4 ± 0.2 |
| ADP | 283 ± 46.2 | 347 ± 51.6 | 402 ± 33.7 | 318 ± 60.1 | 216 ± 24.0 | 245 ± 21.2 | 324 ± 50.2 | 304 ± 25.5 | 282 ± 27.9 | 181 ± 18.4 # |
| GDP | 104 ± 21.6 | 119 ± 20.9 | 131 ± 15.6 | 103 ± 19.7 | 70.2 ± 9.0 | 83.1 ± 10.5 | 112 ± 21.6 | 147 ± 9.9 | 96.8 ± 11.0 | 72.8 ± 7.5 |
| CDP | 27.0 ± 5.4 | 31.9 ± 5.1 | 30.7 ± 3.8 | 26.4 ± 5.5 | 17.1 ± 2.3 | 22.5 ± 2.4 | 33.1 ± 6.9 | 12.4 ± 2.8 § | 21.8 ± 1.9 | 13.8 ± 1.8 # |
| TDP | 1.3 ± 0.2 | 1.1 ± 0.5 | 1.3 ± 0.2 | 1.3 ± 0.4 | 0.5 ± 0.2 | 1.1 ± 0.2 | 1.1 ± 0.4 | 0.7 ± 0.2 § | 0.9 ± 0.2 | 0.3 ± 0.2 # |
| UDP | 68.6 ± 10.5 | 79.9 ± 10.6 | 81.8 ± 7.5 | 60.6 ± 10.3 | 50.0 ± 5.4 | 59.6 ± 5.3 | 76.3 ± 10.7 | 35.7 ± 7.4 † § | 54.0 ± 5.5 | 36.6 ± 3.7 # |
| ATP | 1595 ± 117 | 1890 ± 198 | 1541 ± 126 | 1331 ± 93.5 | 1602 ± 174 | 1656 ± 152 | 1874 ± 183 | 519 ± 235 † § | 1381 ± 88.7 | 1106 ± 130 |
| GTP | 376 ± 20.6 | 452 ± 45.8 | 401 ± 28.2 | 323 ± 30.1 | 357 ± 37.7 | 417 ± 32.4 | 468 ± 36.9 | 173 ± 58 † § | 358 ± 38.0 | 320 ± 30.4 |
| CTP | 88.4 ± 5.7 | 108 ± 11.0 | 84.6 ± 8.8 | 73.2 ± 4.7 | 83.9 ± 9.5 | 96.5 ± 8.2 | 113 ± 11.3 | 24.7 ± 16.3 † § | 77.0 ± 6.4 | 53.5 ± 4.6 # |
| TTP | 2.9 ± 0.6 | 3.5 ± 0.4 | 2.9 ± 0.5 | 2.1 ± 0.2 | 1.8 ± 0.3 | 3.6 ± 0.5 | 3.5 ± 0.5 | 1.0 ± 0.6 † § | 1.9 ± 0.3 | 1.8 ± 0.3 |
| UTP | 228 ± 17.0 | 251 ± 23 | 188 ± 21.1 | 149 ± 9.3 | 204 ± 23.9 | 249 ± 21.4 | 254 ± 28.3 | 51.8 ± 34.1 † § | 152 ± 12.7 | 125 ± 13.8 |
| dATP | 12.8 ± 1.1 | 14.9 ± 1.6 | 12.4 ± 1.0 | 10.8 ± 0.7 | 13.0 ± 1.2 | 13.6 ± 1.1 | 14.4 ± 1.9 | 4.5 ± 2.0 † § | 11.6 ± 0.8 | 9.9 ± 1.1 |
| dCTP | 2.1 ± 0.2 | 2.8 ± 0.5 | 2.0 ± 0.2 | 1.6 ± 0.3 | 1.7 ± 0.2 | 2.7 ± 0.3 | 3.4 ± 0.8 | 0.7 ± 0.3 † § | 1.6 ± 0.2 | 1.3 ± 0.3 |
| Hypoxanthine | 0.7 ± 0.5 | 1.5 ± 0.7 | 5.0 ± 1.6 | 5.2 ± 0.4 | 1.7 ± 0.6 # | 0.9 ± 0.9 | 2.0 ± 0.7 | 132 ± 34.0 † § | 2.7 ± 1.1 | 0.6 ± 0.4 |
| Xanthine | 12.6 ± 0.8 | 12.7 ± 0.8 | 18.4 ± 1.0 † | 21.7 ± 1.1 | 19.8 ± 1.0 | 14.0 ± 1.1 | 11.1 ± 1.0 | 54.0 ± 8.9 † § | 21.4 ± 1.4 | 22.7 ± 2.2 |
| NAD | 111 ± 10.5 | 140 ± 8.9 | 68.6 ± 2.0 † | 56.2 ± 10.8 | 53.7 ± 5.1 | 108 ± 8.7 | 144 ± 17.8 | 56.7 ± 3.4 † § | 52.9 ± 4.3 | 46.8 ± 7.0 |
| NADH | 14.7 ± 3.3 | 13.9 ± 3.2 | 56.2 ± 11.3 † | 48.0 ± 8.7 | 38.6 ± 6.7 | 20.0 ± 4.1 | 14.5 ± 2.4 | 40.1 ± 4.2 † | 49.8 ± 10.1 | 42.5 ± 7.6 |
| NADP | 9.3 ± 1.4 | 10.7 ± 1.2 | 7.3 ± 0.6 † | 6.1 ± 1.6 | 4.7 ± 0.5 | 8.9 ± 0.8 | 12.3 ± 2.6 | 4.3 ± 0.8 † § | 5.1 ± 0.7 | 4.3 ± 0.6 |
| FAD | 4.1 ± 0.3 | 4.1 ± 0.4 | 4.1 ± 0.2 | 4.1 ± 0.3 | 4.0 ± 0.2 | 4.2 ± 0.3 | 4.8 ± 0.4 | 3.5 ± 0.2 † | 4.1 ± 0.3 | 3.6 ± 0.2 |
| Energy Charge (ATP+1/2ADP)/(ATP+ADP+AMP) | 0.84 ± 0.05 | 0.83 ± 0.05 | 0.81 ± 0.04 | 0.84 ± 0.03 | 0.88 ± 0.03 | 0.88 ± 0.03 | 0.85 ± 0.05 | 0.48 ± 0.10 † | 0.87 ± 0.01 | 0.86 ± 0.03 |

| | Contralateral | | | | | Ipsilateral | | | | |
|---|---|---|---|---|---|---|---|---|---|---|
| | Normal (5) | Sham (5) | Hypoxia-Ischemia (5) | Normothermia (7) | Hypothermia (8) | Normal (5) | Sham (5) | Hypoxia-Ischemia (5) | Normothermia (7) | Hypothermia (8) |
| **Purine Pyrimidine metabolism** | | | | | | | | | | |
| Allantoin | 16.4 ± 4.4 | 24.7 ± 3.2 | 48.2 ± 8.4 | 81.7 ± 4.5 | 39.3 ± 3.2 # | 20.9 ± 1.4 | 22.4 ± 2.8 | 38.6 ± 5.1 † | 86.3 ± 8.5 | 49.3 ± 6.3 # |
| β-Ala | 21.5 ± 1.2 | 23.7 ± 2.2 | 23.3 ± 2.3 | 21.5 ± 2.0 | 21.1 ± 1.6 | 23.8 ± 1.2 | 23.0 ± 1.4 | 23.4 ± 1.7 | 21.6 ± 1.7 | 22.3 ± 1.2 |
| **Fatty acid metabolism** | | | | | | | | | | |
| L-Carnitine | 63.1 ± 3.2 | 56.3 ± 2.4 | 33.6 ± 2.0 † | 57.2 ± 3.8 | 72.2 ± 3.8 # | 66.4 ± 4.4 | 58.6 ± 0.9 | 24.4 ± 2.8 † § | 57.4 ± 3.8 | 65.6 ± 3.6 |
| β-Hydroxybutyrate | 149 ± 14.7 | 205 ± 26.9 | 915 ± 69.8 † | 300 ± 38.8 | 241 ± 13.0 | 158 ± 13.2 | 204 ± 13.7 * | 1124 ± 131 † | 331 ± 55.7 | 268 ± 11.1 |
| Malonyl CoA | 507 ± 39.3 | 523 ± 54.2 | 525 ± 41.7 | 576 ± 35.0 | 723 ± 50.7 # | 571 ± 39.1 | 546 ± 50.6 | 202 ± 103 † § | 579 ± 26.7 | 488 ± 34.8 |
| **Glycogen / Glucuronate metabolism** | | | | | | | | | | |
| UDP-glucose | 149 ± 8.8 | 203 ± 15.8 * | 123 ± 2.9 † | 102 ± 17.0 | 69.9 ± 5.7 | 160 ± 12.3 | 209 ± 20.1 | 55.6 ± 16.7 † § | 87.5 ± 8.3 | 47.2 ± 5.5 # |
| **Hexose metabolism** | | | | | | | | | | |
| UDP-N-acetylglucosamine | 212 ± 4.2 | 227 ± 10.4 | 183 ± 8.5 † | 188 ± 7.1 | 183 ± 5.6 | 217 ± 7.2 | 232 ± 4.0 | 98.6 ± 20.9 † § | 173 ± 9.4 | 126 ± 5.4 # |
| **Miscellaneous** | | | | | | | | | | |
| Carnosine | 3.9 ± 1.9 | 4.0 ± 0.8 | 1.0 ± 0.3 † | 7.1 ± 2.5 | 2.8 ± 0.9 | 3.6 ± 1.2 | 3.8 ± 1.1 | 2.2 ± 0.8 | 6.2 ± 2.1 | 3.8 ± 1.3 |
| 2-Aminobutyrate | 24.2 ± 2.3 | 23.9 ± 1.3 | 65.3 ± 4.6 † | 75.2 ± 4.1 | 59.8 ± 5.1 # | 25.5 ± 2.2 | 24.8 ± 0.8 | 56.1 ± 3.5 † | 91.3 ± 7.7 | 83.8 ± 7.3 |
| 2-Hydroxyglutaric Acid | 23.5 ± 1.0 | 31.3 ± 1.8 * | 56.7 ± 2.8 † | 33.5 ± 3.7 | 24.4 ± 1.4 | 23.1 ± 1.4 | 30.2 ± 0.8 | 32.9 ± 5.8 § | 30.9 ± 5.0 | 15.8 ± 1.0 # |
| **Neurotransmitter** | | | | | | | | | | |
| Acetylcholine | 1.1 ± 0.1 | 1.0 ± 0.2 | 1.4 ± 0.3 | 0.8 ± 0.1 | 0.5 ± 0.1 # | 0.9 ± 0.1 | 0.8 ± 0.1 | 2.5 ± 0.4 † | 0.8 ± 0.2 | 0.6 ± 0.1 |
| Choline | 70.3 ± 6.5 | 49.9 ± 3.6 * | 45.1 ± 4.4 | 53.6 ± 5.1 | 61.0 ± 4.7 | 66.9 ± 2.7 | 47.3 ± 2.5 * | 162 ± 31.6 † § | 52.9 ± 2.4 | 90.7 ± 7.4 # |

Values are means ± SEM, in nmol/g tissue, from measurements made in the number of animals indicated in the parentheses. *, † and #: $P < 0.05$ compared with the normal, the sham and the normothermia, respectively. §: $P < 0.05$ compared with the hypoxia-ischemia on the contralateral hemisphere. Differences between the means were evaluated for significance using Student's *t*-test. N.D.; not detected.

## Fig. S1

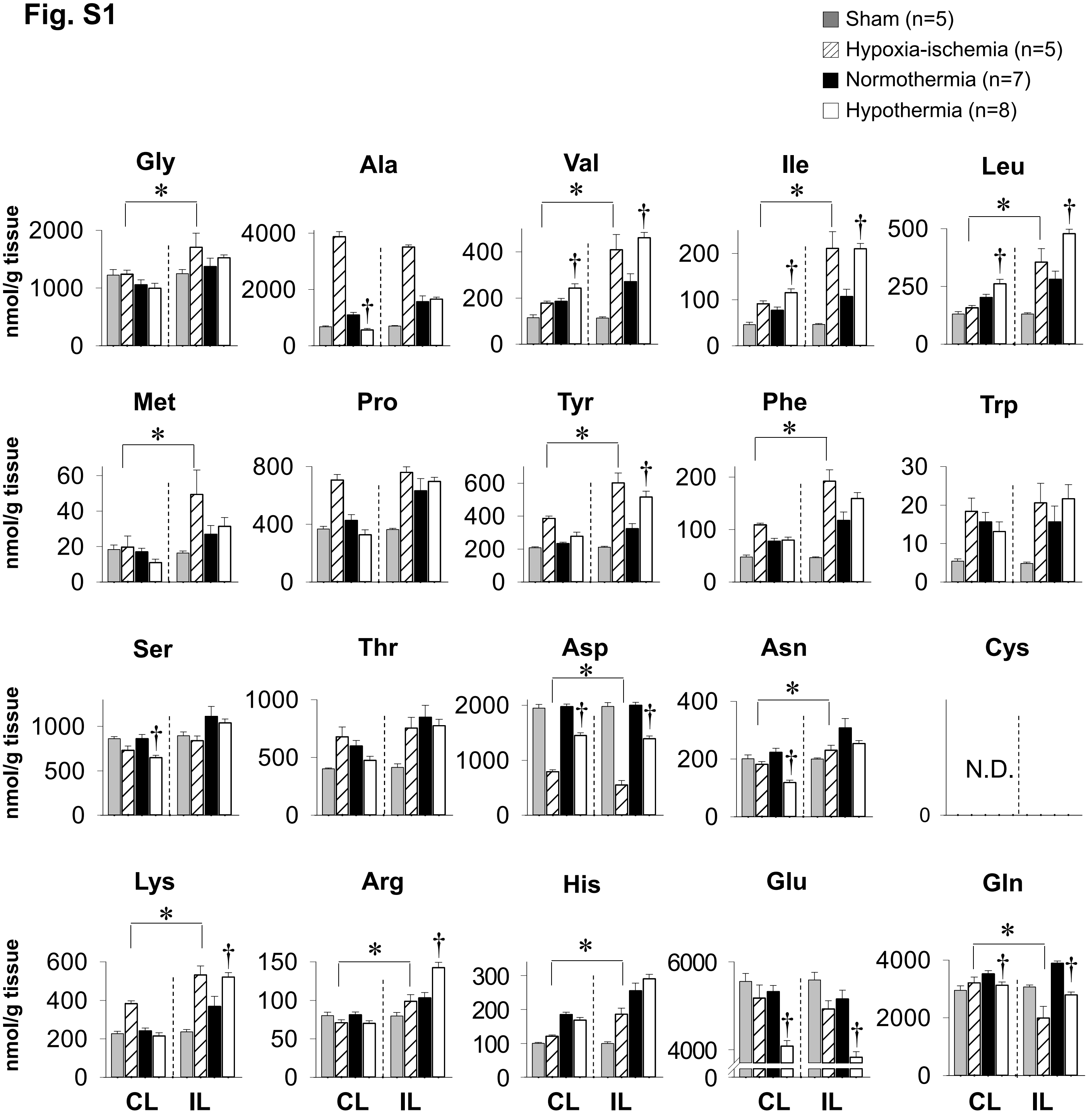


**Figure S1. Amino-acid metabolome.** Contents of standard amino acids determined using capillary electrophoresis electrospray ionization mass spectrometry (CE/ESI/MS). CL; contralateral hemisphere, IL; ipsilateral hemisphere. * and †: $P < 0.05$, compared with the CL, and the normothermia, respectively. Differences between the means were evaluated for significance using Student's *t*-test. N.D., not detected.

**Fig. S2**

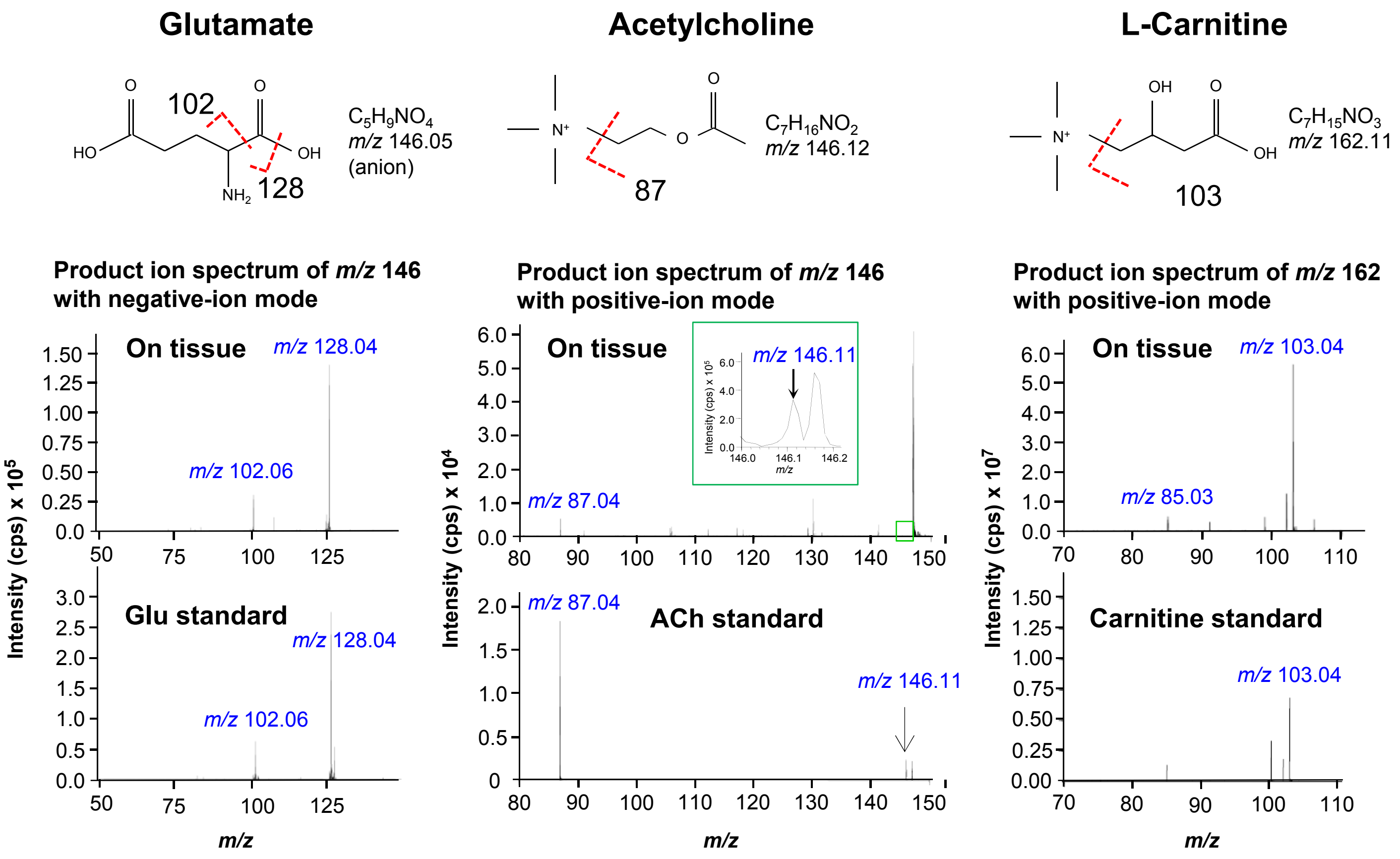


**Figure S2. Tandem MS analysis to identify glutamate, acetylcholine and carnitine.** The chemical structures show the assignments of the diagnostic fragments. Comparisons of tissue MS/MS spectra with ion peaks at *m/z* 146 (negative-ion mode), *m/z* 146 (positive-ion mode) and 162 (positive-ion mode) (*top*) and authentic glutamate, acetylcholine and carnitine (*bottom*), respectively. The similarity of the two spectra was used to assign the metabolites as glutamate, acetylcholine and carnitine.
